# The alien slipper limpet *Crepipatella dilatata* (Lamarck, 1819) in northern Spain: A multidisciplinary approach to its taxonomic identification and invasive biology

**DOI:** 10.1371/journal.pone.0205739

**Published:** 2018-10-30

**Authors:** Alexandra Richter, Alberto M. Gándara, Francisco Silva, Antonio Brante

**Affiliations:** 1 Department of Biology of Organisms and Systems, Faculty of Biology, University of Oviedo, Oviedo, Spain; 2 Grigore Antipa National Museum of Natural History, Bucharest, Romania; 3 Department of Molecular Biology, University of Bucharest, Bucharest, Romania; 4 Department of Ecology, Universidad Católica de la Santísima Concepción, Concepción, Chile; 5 Centro de Investigación en Biodiversidad y Ambientes Sustentables (CIBAS), Universidad Católica de la Santísima Concepción, Concepción, Chile; Laboratoire de Biologie du Développement de Villefranche-sur-Mer, FRANCE

## Abstract

The slipper limpet *Crepipatella dilatata*, native to Chile and Argentina, was introduced in Spain in 2005. The species was thought to inhabit the region of Rias Bajas, yet recently, putative *C*. *dilatata* populations have been documented on the coast of north-central Spain and in the Ebro Delta of the Spanish Mediterranean. Here we undertook a multidisciplinary approach to study the invasion biology of this species. Specifically, two geographically distant populations, one being a successfully established population from O Grove and the other a declining population from Gijon, were studied over the course of four years. Analyses of morphological and developmental traits as well as genetic information confirmed the presence of *C*. *dilatata* in these sites. The results revealed polymorphism in anatomical traits and shell shape. Shell shape polymorphism was unevenly distributed among sites and among sexes. Males were monomorphic, while females were polymorphic. Of the female morphotypes encountered, one was absent in the declining population from Gijón. Size at first female maturation and female size were greater in the declining population than in the established population. Reproductive success varied seasonally but not spatially among populations. In the established population, gregariousness was significantly greater; the size when sex changes was found to be plastic and socially controlled. The sex ratio of the declining population was female biased while in the established population the sex ratio changed during the study period from being balanced to being female biased. This change in sex ratio was probably due to higher male mortality. Molecular analyses pointed to the localities of Corral Bay in southern Chile and Puerto Madryn in southern Argentina as potential population sources. The intercontinental import of fresh mussels cultivated in Chilean farms is a likely source of this mussel in Spain. Comparison with available data of native populations of *C*. *dilatata* strongly indicate that ecophenotypic plasticity, socially controlled sex change, high gregariousness, increased nurse egg supply to viable larvae during the encapsulated developmental period, later maturation and larger female sizes altogether enhance establishment success of this non-indigenous species. Human-mediated factors like the intraregional mussel trade and transplantation are also likely secondary dispersal mechanisms favouring the spread of this organism.

## Introduction

Non-indigenous species (NIS) transported across natural biogeographical barriers and released, whether unintentionally or deliberately, into new environments have the potential to become invasive species and profoundly alter native biodiversity [1–4]. In turn, invasive species can have adverse and sometimes severe consequences on human health and socio-economical welfare. Given that eliminating marine NIS is extremely difficult once establishment takes place [5, 6], the implementation of policies to prevent further introductions or to prevent and reduce the establishment success of previously introduced NIS are the most effective way to avoid future impacts on native biota [5]. However, in order for policies to be effective, conservation management must have an accurate record of the native biota and must be able to trace and manage potential introduction pathways and vectors [7, 8].

To understand invasions and to develop methods of invasion control it is also necessary to identify the factors, and their interactions, that promote establishment success and the spread of NIS [9]. Potential invaders that are released to the receptor environment and initiate the invasion process are exposed to a series of filters that operate at different stages in the progression of the invasion: introduction, establishment and spread [10]. As such, NIS may be successful or fail in passing from one stage to the next. In particular, during the first stages of the invasion process, when population sizes are generally low, founder and Allee effects may impede NIS to establish successfully, grow demographically, and spread [9]. Overall, many interacting factors, such as asexual reproduction [9, 11], plasticity in life history traits [12, 13, 14], hermaphroditism [9, 11] and gregariousness [11, 15], likely influence invasion success.

The ability to reproduce asexually, widespread among many successful invaders, can allow organisms to avoid Allee effects as well as inbreeding depression [9, 11]. Its role in the invasion success becomes evident in invasive species with flexible reproductive modes that switch from a predominantly sexually reproductive mode in the native range of distribution to a widespread asexual reproduction following introduction [9, 11, 16]. Obligately sexual species may present other mechanisms to overcome founder effects. For example, gregarious behaviour facilitates sexual encounters for individuals with internal fertilization or may favour sperm-ovum encounters for free spawning organisms [11, 15, 17]. Simultaneous hermaphrodites may double the number of mating partners [11]. Additionally, the timing of sex change of some sequential hermaphrodites has been shown to be plastic with sex change delayed or advanced according to the local social configuration [18–21], a strategy that maximizes mating and individual reproductive success [17, 22, 23]. Changes in the social structure of populations are relevant to the success of the post introduction stages of invasion in that they are correlated with increased ability for resource exploitation and competitive success [24, 25]. Thus, studying the life history traits, social behaviour, and reproductive strategies of NIS in receptor environments can inform how a particular species will respond to a new environment and can be useful for predicting invasion success.

Molluscs are one of the groups of non-indigenous marine organisms most frequently introduced to coastal and estuarine ecosystems [26–29]. Their main introduction pathways are shipping and aquaculture activities, which often include intentional introductions as exotic commodities as well as accidental introductions as biofouling or accompanying species [27, 29–31]. These NIS include members of the gastropod family Calyptraeidae, commonly known as slipper limpets. Calyptraeids are sessile filter-feeders that live as epibionts on barnacles, kelp holdfasts, gastropods and bivalves or attached to hard substrata such as rocky boulders and the empty shells of bivalves and gastropods [21, 32–35]. They are protandric hermaphrodites with a mobile male phase and a sessile female phase; the female incubates egg capsules under her shell between the foot and the mantle [17, 32–37]. With a few exceptions [38–40], slipper limpets tend to be gregarious to variable degrees [15, 17, 18, 20, 21, 39]. Field and experimental data have also shown growth rate, sexual maturity and size at sex change to be highly plastic in this group, and that local social interactions are important factors explaining this plasticity [17, 18, 20, 21, 23]. Developmental strategies are generally species-specific and may range from planktotrophic development with females producing thousands of planktonic feeding larvae, to non-planktotrophic development in which offspring hatch as benthic juveniles or pediveligers after consuming yolk, nurse eggs, or other embryos [36]. Although not all known introduced slipper limpets have become invasive [30, 41–43], their hermaphroditic nature, the trend in forming aggregations together with the high dispersal potential or the low mortality risk of early life stages in species with non-planktotrophic development, make members of this group excellent candidates as successful invaders.

To date three non-indigenous slipper limpets are present in the Iberian Peninsula: *Bostrycapulus odites*, *Crepidula fornicata*, and *Crepipatella dilatata*. *Bostrycapulus odites* originates from the Southwest Atlantic and currently has a very restricted distribution in the Iberian Peninsula [42, 43]. *Crepidula fornicata* is considered an invasive species with well established populations along the Atlantic coast of northern Spain and is native to the Atlantic coast of North America [31, 44–46]. Lastly, *Crepipatella dilatata* is native to the coasts of Chile and Argentina [32–34, 37] and belongs to a complex of cryptic species that includes at least two other species not yet known as NIS: *Crepipatella fecunda* and *Crepipatella occulta* [33, 37, 40]. Additionally, *C*. *dilatata* lives in sympatry in Coquimbo, northern Chile, with *C*. *occulta* and both share the non-planktotrophic developmental mode [37, 40]. Individuals with the morphotype characteristic of *C*. *dilatata* were detected for the first time in Galicia (NW Spain) in 2005 attached to bivalves dredged from the Aldán estuary [47]. Between then and 2014, the number of new records of this species along the Spanish coast has increased. In Galicia, the range of *C*. *dilatata* has expanded along the coast of the Rias Bajas colonizing the estuaries of Vigo, Pontevedra, and Arousa [47, 48]. In the Arousa estuary, this species has considerably impacted the infrastructure of shellfish cleansing systems [48]. Despite this, Galician populations have not been monitored, and evidence of juvenile recruitment and self-sustained reproduction has not been provided. In 2010, a reproductive population of *C*. *dilatata* was reported close to the commercial port of Gijon on the central Cantabrian coast (North Spain) [49, 50], and more recently, in 2014, several individuals were found in the Ebro Delta on the Mediterranean coast of Spain [51]. However, most of these reports of *C*. *dilatata* in Galicia and in the Ebro Delta involved only taxonomic identification using shell morphological traits, which have been shown to be unreliable given the existence of cryptic species. Currently, the taxonomic identity of only a single population from Beluso bay, in the Pontevedra Estuary (Galicia, NW Spain), has been verified with developmental traits and with molecular barcoding tools [52].

Source of origin as well as the vectors and pathways of the first introduction of *C*. *dilatata* in Galicia are still unresolved [47, 52]. Through DNA barcoding, Collin et al. [52] revealed three haplotypes of Spanish specimens that matched with natives ones distributed through Central Chile. Yet sample size used were too small to determine the exact source of origin of the introduced populations and discard Argentine as potential source of origin [52]. Post-border dispersal mechanisms favoring the rapid spread of *C*. *dilatata* in the Rias Bajas are also unknown. Collin et al. [52] suggested that mussel farming might have contributed to the intraregional, post-border dispersal of *C*. *dilatata* in NW Spain [52]; however, conclusive evidence for this is lacking to date.

In the present study, two geographically distant populations of *C*. *dilatata*, one that has failed to become reproductively self-sustaining and another that is successfully self-replenishing, were monitored over a period of four to six years. The main objectives were: (a) firstly, using an integrative taxonomic approach based on phenotypic traits (anatomy, radula and shell morphology) and genetic data, to unambiguously determine the taxonomic status of samples taken from the two sites (b) secondly, to assess the intraspecific variation in social behaviour, population characteristics, and life history traits (adult body size, size at sex change, and reproductive success) as potential driving factors of invasion success (c) lastly, to shed light on the potential origin and vector of first introduction and secondary dispersal mechanism.

## Material and methods

### Ethical statement

The organism of interest in the present study is a South American marine gastropod that has been introduced by man outside its native distribution range to the Iberian Peninsula. In Europe, this NIS is not commercially exploited as a marine resource nor is it legally protected. The sites where most of the individuals were collected are highly used beaches where sampling of organisms not legally protected does not require any special permits from the local authorities. For this study, many samples were obtained from fresh native mussels sold in the retail market (*C*. *dilatata* is a fouling organism) and from mussels attached to discarded ropes found in the sea. Voucher specimens used here for the planned analyses and from which tissue samples were obtained for DNA extraction and barcoding have been deposited in three public repositories, the Malacological Collection of the National Museum of Natural Sciences of Madrid, Spain (MNCN), the Zooarchaelogical collection of the University Autónoma of Madrid (ZA) and the Zoological Collections of the Department of Biology of Organisms and Systems (BOS) at the University of Oviedo. The remainder of the samples were labelled with identification codes (cd) and deposited in the BOS department or are under custody of the first author (A. Richter) pending transfer to public repositories.

### Samples

Two sites from north Spain, an exposed rocky shore in O Grove (Galicia, NW Spain) and an artificial sandy beach in Gijon (Asturias, central northern Spain), El Arbeyal, where individuals of the morphotype of *C*. *dilatata* had been previously recorded, were sampled twice in a time span of four years, once in autumn of 2012 and once in spring of 2016. O Grove is located on a small peninsula in the Arousa estuary at the entrance of O Grove Bay. This bay, an important shellfish production and manufacturing centre due to the high productivity of the seagrass meadows of *Zostera marina* and *Z*. *noltei* [53], harbours several non-indigenous marine molluscs mostly introduced via aquaculture activities [46, 53, 54]. Approximately 74% of the national production of the native mussel *M*. *galloprovincialis* occurs on floating rafts close to the shoreline of this bay [55]. The lower intertidal fringe composed of natural bedrock and artificial hard substrate is covered by mussel beds of *M*. *galloprovincialis*, which may also form loose clumps scattered on the soft bottom of the bay.

El Arbeyal (43° 32’N, 5° 39’W), the other study site, is a small sheltered artificial sandy beach close to the commercial port of El Musel in Gijon. The intertidal of this beach, strongly altered by human activities, is a sandy-muddy flat with scattered rocky boulders and patches of soft sedimentary rock that harbours the common piddock *Pholas dactylus*, cup oysters (*Crassostrea* sp.) and scattered aggregates of native *M*. *galloprovincialis* mussels. As in O Grove, native mussels also form small clumps lying unattached on the sea bottom and form small mussel beds on artificial substrata.

In both sites, sampling was conducted during low tide in the intertidal zone by inspecting potential substrata: scattered stones, rocky boulders, natural mussel beds, wild mussels on artificial substrata, unattached mussel clumps, and empty bivalve shells. *Crassostrea* sp. cup oysters on hard artificial substrata were also surveyed in El Arbeyal in autumn 2012 and April 2016. Mussels attached to stranded culture ropes cast off to sea in April 2016 in O Grove and retail Galician mussels acquired between 2013 and 2016 were also checked for attached slipper limpets. Galicia has an important market of fresh *Mytilus galloprovincialis* mussels cultured in southern Chilean farms [56]. This together with the fact that *C*. *dilatata* is a mussel epibiont in its native range [33, 52, 57] makes farmed Galician mussels suceptible to be infested by this NIS and be used by it as potential post-border dispersal vector. Specimens with the *C*. *dilatata* morphotype (benthic individuals and broods) collected in April 2010 at El Arbeyal, directly preserved in 70% ethanol, and deposited in the BOS collection were also re-examined to corroborate taxonomic identification and for use in further analyses.

All specimens collected between 2012 and 2016 were brought to the laboratory and processed. Living animals were fixed in a 4% formalin solution with seawater and, except for the egg-masses, were later preserved together with their shells in 70% ethanol for subsequent studies. Prior to fixation, fresh tissue samples were extracted from the foot muscle of individuals selected for the DNA barcoding analysis and preserved in 95% ethanol. Dead animals were discarded and only their shells were preserved dry or in ethanol. The tissue samples from 2010 deposited in the BOS collection and used for the molecular analyses were obtained from previously preserved specimens. For the shell morphological analysis, additional intact empty shells of freshly dead specimens were randomly collected from the intertidal of O Grove in April 2016. All the relevant information of the material collected and studied is summarised in Table 1.

**Table 1:** *Crepitapella dilatata* samples used in the present study.

Source	Sampling site/ location of mussel culture rafts	Date of collection	N	Substrata	Vouchers and identity codes
**Field sampling**	El Arbeyal	April 2010	47 individuals	Stones; loose clumps of native mussels (*M*. *galloprovincialis*) falling within the size range of marketed mussels (shell length: 40.0–85.8 mm)	BOS-GAS 110; BOS-GAS111; BOS-GAS 113; BOS-GAS 114; BOS-GAS 123–130; BOS-GAS 145–156; BOS-GAS 158; MNCN 15.05/75.175; MNCN 15.05/75.176
**Field sampling**	El Arbeyal	October 2012	3 individuals	Native Mussels (*M*. *galloprovincialis*) on artificial substrata	BOS-GAS 112; BOS-GAS 144; BOS-GAS 158
**Field sampling**	O Grove	September 2012	100 individuals	Stones; rocky boulders; native mussels (*M*. *galloprovincialis*); living periwinkle (*Littorina littorea*)	BOS-GAS 135–138; MNCN 15.05/75170-MNCN 15.05/75174; cd-30-61; cd-64; cd-66-76; cd-78-140;
**Field sampling**	O Grove	April 2016	28 individuals + 43 shells	Native mussels on culture ropes; unattached native mussel clumps; dead shells of *R*. *philippinarum* and *Lutraria oblonga*; stones	MNCN 15.05/78298; ZA CRE DIL 2–3; ZA CRE DIL 5–7; cd-141-166
**Retail market in Oviedo**	Cambados	November 2013	1 individual	fresh marketed native mussels cultured on floating rafts	cd-169
**Retail market in Madrid**	Galicia	January 2014	1 individual	Fresh marketed native mussels cultured on floating rafts	cd-170
**Retail market in Oviedo**	Galicia	September 2015	1 individual	Fresh marketed native mussels cultured on floating rafts	private collection A. Richter (01/00.022)
**Restaurant/Wholesaler Amegrove**	O Grove	April 2016	1 shell	Fresh marketed native mussels cultured on floating rafts	ZA CRE DIL 4

Abbreviations: BOS-GAS, gastropods from the zoological collections of the Department of Biology of Organisms and Systems at the University of Oviedo. N, sample size

### Determination of sexual categories

Sex identification was carried out to ascertain the presence/absence of egg capsules, the grade of development of the gonad and secondary sexual characters. Individuals lacking egg capsules, with seminal vesicles and a long well-developed penis were identified as male. Females were identified as individuals incubating egg-capsules or with a completely developed functional female pallial reproductive system consisting of an albumen gland with associated seminal receptacles, a capsule gland, a female genital papilla, and either with or without a reduced penis. Individuals without gonads and lacking secondary sexual characters were considered juveniles. Individuals with a reduced penis and an incompletely developed female pallial reproductive system were classified as intersex stages. As size variable we used shell length (SL). In calyptraeids, due to their epibiotic life-style, this shell dimension corresponds to the length of their shell aperture (see below), whose margin fits tightly to the surface of the host or substratum. For most individuals, SL was measured with a calliper to the nearest 0.05 mm. Small individuals (SL < 4 mm) were measured under a dissecting microscope using an ocular micrometer.

### Anatomical analysis

A set of individuals from each site and from the mussels sold in the retail market were dissected under a stereomicroscope in order to inspect the external and internal anatomy in more detail. In 12 of these individuals (SL: 14.5–36.3 mm), including males and females from both sites, the radulae were extracted from the buccal bulb, cleaned by boiling them gently in a concentrated solution of NaOH, rinsed in distilled water, dehydrated in 70% ethanol, mounted in Hoyer’s Solution on a slide and examined with an optical microscope. Several radulae were also prepared for examination with scanning electron microscopy (SEM). For that, cleaned radulae were transferred to acetone and mounted on an aluminium stub. After allowing the samples to air dry, they were sputter-coated with gold and analysed in a high vacuum and micrographed with a JEOL 6610 LV scanning electron microscope. The analysis of the soft body and radulae were used to complement other taxonomical criteria used in this study (shell morphology, developmental mode, and DNA information).

### Shell morphology

In the native distribution range, *C*. *dilatata* exhibits variability in the shell morphology [32, 37] and the shell of some specimens are almost impossible to distinguish from those of its cryptic species *C*. *occulta* [37]. In addition, studies on non-indigenous gastropods have reported a high morphological variation of certain shell traits in post-introduction stages [16]. Therefore, to determine unambiguosly the taxonomic identity of *C*. *dilatata*, a quantitative morphological analysis of the shell of non-native specimens is helpful. It may be used as reference for future comparative studies. In addition, it may allow tracking temporal and/or spatial changes in the variability of phenotypic traits in postintroduction stages of the invasion process.

**Fig 1:**
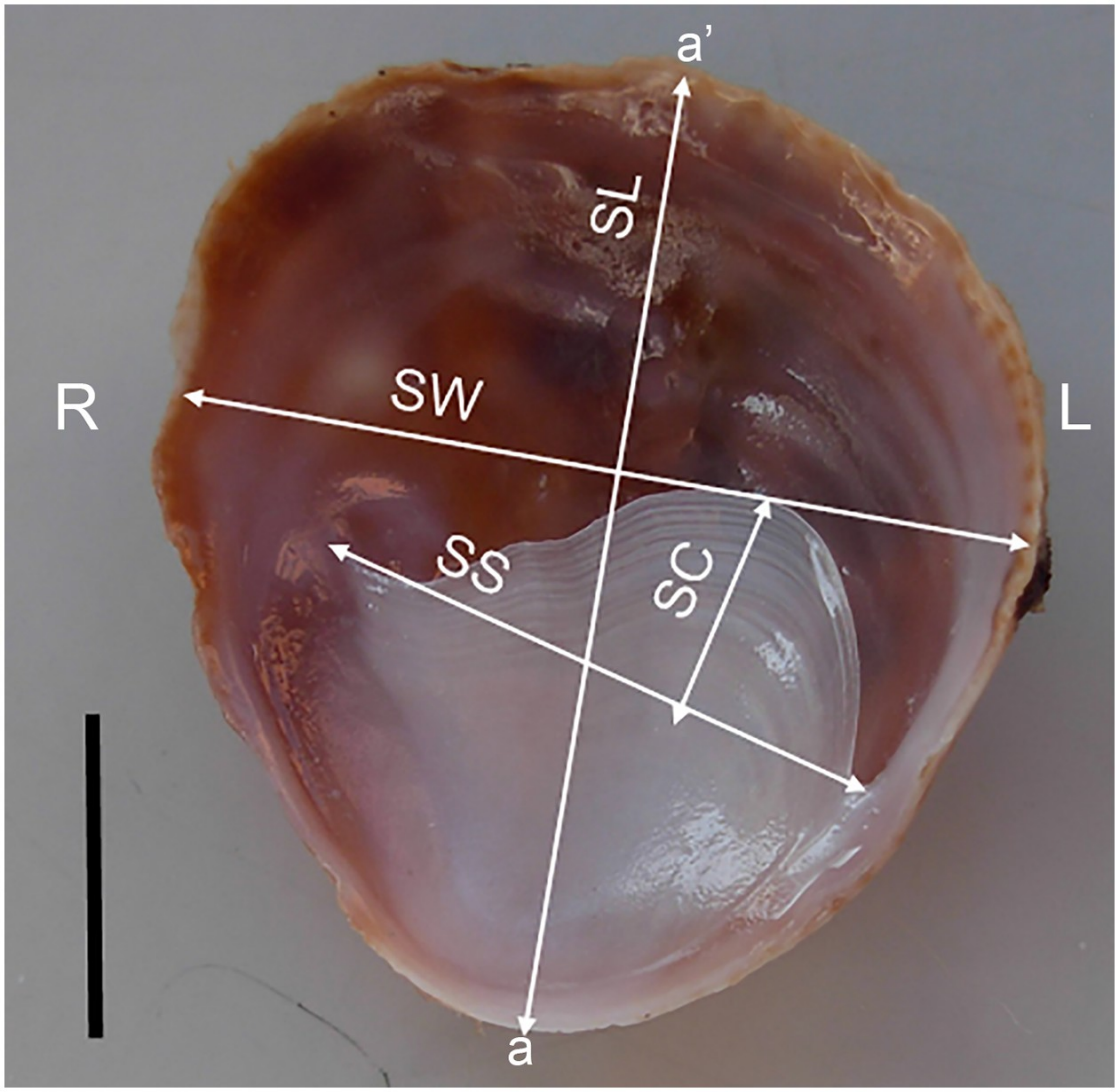
Apertural view of a *Crepipatella dilatata* morphotype IV shell showing the four linear shell dimensions measured for the PCA. Abbreviations: a-a’, antero-posterior axis; L, left side of the shell; R, right side of the shell; SC, height of the septum curvature; SL, shell length; SS, width of the shell septum; SW, shell width. Scale bar: 1 cm.

Thus, a total of 59 shells of *C*. *dilatata*, subsampled randomly from the material collected from O Grove and El Arbeyal, were photographed and digitalized for morphometric analyses. The SL of these samples ranged from 7.55 mm to 43.8 mm including male, intersex, and female individuals. For intact shells collected in April 2016 from O Grove from recently dead specimens, sex was inferred by comparing SL with the size-gender distribution of the population for that year. Shells were sexed as males if SL was within the size range of males (7.55 mm ≤ SL ≤ 12.25 mm; see below) and as females if SL was larger than the maximum size of the intersex (SL > 20.10 mm; see below). Four linear shell dimensions, SL, shell width (SW), width of the shell septum (SS), and maximum height of the septum curvature (SC) were measured on scaled images (Fig 1). In addition, four non-dimensional variables derived from the linear shell dimensions and representing shell aperture shape (SW/SL) and septal relative size (SC/SW and SC/SL) and shape (SC/SS) were calculated for each shell (S1 Dataset). The values of these linear shell dimensions and non-dimensional shell variables were standardised to avoid scaling effects by substracting from the observed values the sample mean and dividing the obtained difference by the sample SD [58]. Then, a Principal Component Analysis (PCA) was combined with a k-means clustering analysis in order to explore eventual morphological patterns (morphotypes) contained in the shell variability of the sample. All standardised variables except SC were used. The latter variable was excluded from both analyses, because after standardisation it did not render a normal distribution.

In this exploration, we ran first the PCA with the complete dataset to extract all those principal components (PCs) with an eigenvalue equal to or larger than 1 and reduce the hyperspace of shell variability into a smaller n-dimensional space defined by these PCs (dimensions). As the PCA retained three PCs (S1 Table), we then set the number k of clusters in the subsequent k-means clustering analysis to 4 using the same set of standardised variables. We proceeded in this way to delimit the morphotypes of our observed shell variability, because the k number of clusters recovered in a reduced hyperspace of n dimensions (PCs) is k = n +1 [59]. The members of the clusters revealed by the k-means clustering (S1 Dataset) were then plotted onto the bi-dimensional space defined by the first two PCs. For purporses of simplifying the present and eventual future descriptive and comparative studies of *C*. *dilatata*’s shell morphology, a General Linear Model (GLM) analysis of variance was in addition conducted to establish differences among the four morphotypes only in those explanatory variables contributing most to the variance of each extracted PC; this was done in STATISTICA 8.0 [60]. An analysis of co-variance was run with SL as the continuous predictor (= co-variable) and “cluster” as the fixed categorical factor. The effects of the independent explanatory variables, the co-variable and the categorical factor were tested for significance following a full factorial design. An *a posteriori* Tukey test for unequal sample sizes (unequal N HSD) was then applied to detect differences among all pairs. For the GLM analysis, the untransformed variables were used after verifying homoscedasticity via Hartley’s *F*max-test, and the normality of the residuals was checked by means of a normal probability plot and/or a Chi-square test. A *G*-test of independence [61] was performed to check whether the frequency distribution of the different shell morphs depended on sexual category or site. The William’s correction factor was applied to compute an adjusted *G* statistic (*Gadj*) for the global analysis and all pair-wise comparisons [61].

### Population and life history traits

The population sex ratio and size at sex change in the field for the samples collected in autumn 2012 and spring 2016 from O Grove and in spring 2010 from El Arbeyal were calculated. Sex ratios were expressed as the total number of males divided by the total number of females, and the deviation of these ratios from the Fisherian sex ratio for dioecious species was tested using the χ^2^ statistic adjusted with the Yates correction for continuity [61]. Size at sex change in the field was defined as the SL at which 50% of the individuals in the population were mature females (L_50_) [21, 39]. Estimations of SL_50_ were carried out via logistic regression using logit as the link function and maximum likelihood as the loss function [62]. The size-gender distribution of each population was studied, and size differences among genders within each population and within genders among populations were tested for significance using a one-way Analysis of Variance (ANOVA) when the assumptions for the parametric analysis were met. Fulfilment of the assumptions was checked as described above. For sample sizes smaller than 30, normality was tested using the nonparametric test of Kolmogorov-Smirnov. If homoscedasticity and/or normality of the residuals were not fulfilled, Mann-Whitney U and Kruskal-Wallis nonparametric tests were performed to compare independent groups or for multiple comparisons, respectively.

### Brood traits and reproductive success

Broods from the same samples for which sex ratio was estimated were examined under a dissecting microscope. Mode of intracapsular larval nutrition (i.e. lecithotrophy, adelphophagy, or nurse egg feeding) and the intracapsular larval stages were recorded. Developmental mode was determined on the basis of the larval stage attained by recently hatched offspring and by offspring ready to hatch, after complete consumption of the extra-embryonic yolk supply (nurse eggs). Furthermore, larvae were classified as either planktotrophic, when individuals ready to hatch attained the veliger stage, or non-planktotrophic, when offspring attained the pediveliger or juvenile (crawling) stage.

For each one of these samples, we assessed three variables related to reproductive success: total number of egg-capsules per brood, total number of viable pre-hatching shelled embryos per egg-capsule, and total number of viable pre-hatching shelled embryos per brood. The total number of viable embryos per egg-capsule was directly counted in two egg capsules for each brood, and the total number of viable embryos per brood was estimated by multiplying the mean number of viable embryos per egg-capsule by the total number of egg-capsules per brood. For each variable, we conducted an analysis of co-variance with female SL as the continuous predictor (co-variable) and sample as the fixed categorical factor following a full factorial design. An *a posteriori* Tukey test for unequal sample sizes (unequal N HSD) was run to detect significant differences among pairs. Except for the total number of egg-capsules per brood, both the dependent variables and the continuous predictors were natural logarithm transformed to meet the assumptions of the parametric analysis. Homoscedasticity and normality of the residual distribution was checked as described above. In addition, the total egg content of 2–4 egg-capsules of each brood containing only eggs and/or unshelled embryos in early non-feeding developmental stages was directly counted, and the fecundity per female was estimated by multiplying the total number of egg-capsules in each brood with the mean number of eggs per capsule. An estimate of the fecundity could not be done for specimens from El Arbeyal, because the eggs in each capsule had fixation problems.

### Gregariousness and social control of sex change

To study the gregarious behaviour of *C*. *dilatata* and its influence on size at sex change, group size and composition of aggregates collected from O Grove in autumn 2012 and in spring 2016 and from El Arbeyal in spring 2010 were recorded. For most aggregates from O Grove, which were handled carefully to avoid dislodging and placed separately into individual hermetically sealed plastic bags, group sizes were assessed by directly counting all of the aggregation’s members, and size and sex of individuals were determined directly following the procedure described above. For aggregates collected from El Arbeyal whose members were accidentally dislodged after collection, group size, sex, and size of individuals were inferred indirectly by applying four different criteria: (1) the number and size (maximum diameter) of the home scars left by hitchhikers on the shell of any female individual x_j_ that had served as substrate (focal female), (2) the SL of the smallest female individual with a home scar, (3) presence or absence of egg capsules inside home scars, and (4) the SL range for each sex category observed directly in the population sample. Accordingly, male sex was assigned to home scars ranging from 9.45 to 20.0 mm in maximum diameter and females to home scars larger than 21.9 mm; home scars with a maximum diameter between 20.05 mm and 21.9 mm and without egg-capsules inside were considered as intersex individuals. In two focal individuals with dislodged hitchhikers from the September 2012 O Grove sample, home scars with maximum diameters between 8.0 and 20.25 mm were assigned to males, those between 20.25 and 29.75 mm were assigned to intersex stages, and those larger than 29.75 mm were assigned to females. In concentric home scars, only the size of the outermost scar was considered for sex determination and aggregate size.

To verify whether the intensity of gregariousness was temporally or spatially variable, the *G*-test of independence was performed to check whether or not the frequency distribution of aggregate size was homogenous among sites, within the same site, and among sampling periods [61]. Since different periods were only sampled in O Grove, temporal effects could be tested only in this site. Three categories of group size were considered: 1) couples, 2) aggregates with three members, and 3) aggregates with more than three members [S2 Dataset]. The William’s correction factor was applied to compute an adjusted *G* statistic (*Gadj*) for all pairwise comparisons [61].

In order to explore the effect of social interactions on size at sex change, the relationship between the smallest female and the largest male size in the aggregates was quantified by calculating the Pearson correlation coefficient between them. Significance was also tested. A significant positive correlation was expected if size at sex change was socially regulated [63]. In addition, the functional relationship between the smallest female size and the largest male size in the aggregates was studied by applying the major axis method (MA) of a model II simple linear regression [64]. The slope and the intercept of the regression line were calculated and tested for significance using the lmodel2 package of the free software R 3.0 [64]. A Spearman rank correlation was also used to test whether the size of the smallest female in the aggregate depended on the total number of individuals in the aggregate [19]. To discard the possible effects of a correlation between the size structure of the aggregate’s members and group size, the correlation between the mean size of the individuals in the aggregate and group size was tested with a Spearman rank correlation. For specimens from Gijon, the influence of group size on size at sex change could not be tested due to the small sample size and because group size ranged from two to three individuals and did not have enough categories.

### Genetic identity and potential source population

We undertook a DNA barcoding approach to complement morphological and reproductive analyses for species identification and to determine the most plausible source of introduction of the calyptraeid specimens sampled from the study localities. As genetic marker, we selected the cytochrome *c* oxidase subunit I (COI) gene, because of its significant discriminatory power between species in barcoding studies of calyptraeids [52, 65]. A total of ten COI sequences from non-native individuals collected here, five from each sampling site (Table 2), were analysed phylogenetically together with all of the COI sequences for *C*. *dilatata* and the sister species *Crepipatella peruviana* (formerly *Crepipatella fecunda* Gallardo, 1979) available in GenBank.

**Table 2:** Summary of the live collected and barcoded material deposited in public repositories.

Voucher number	Locality	Genbank access number
MNCN 15.05/75170	Playa de Corveiro, O Grove	KU315455
MNCN 15.05/75171	Playa de Corveiro, O Grove	KU315451
MNCN 15.05/75172	Playa de Corveiro, O Grove	KU315453
MNCN 15.05/75173	Playa de Corveiro, O Grove	KU232553
MNCN 15.05/75174	Playa de Corveiro, O Grove	KU315452
MNCN 15.05/75175	Playa de El Arbeyal, Gijon	KU315458
MNCN 15.05/75176	Playa de El Arbeyal, Gijon	KU315456
BOS-GAS 128	Playa de El Arbeyal, Gijon	KU315454
BOS-GAS 129	Playa de El Arbeyal, Gijon	KU232554
BOS-GAS 130	Playa de El Arbeyal, Gijon	KU315457

The COI sequences of the non-native specimens were obtained by extracting total genomic DNA from samples of their foot muscle tissue following the salting-out protocol [66]. The COI gene was amplified using the universal primers HCO2198 and LCO1490 [67] in 30 μL reactions containing 3 μL of PCR buffer (10X), 3.6 μL MgCl_2_ (25 mM), 1.5 μL dNTPs (2.5 mM), 0.3 μL BSA (100X), 0.3 μL of each primer (10 μM), 0.25 μL Taq polymerase TopTaq (Quiagen, 5 U μL^-1^) and 3 μL of genomic DNA diluted 1/25. PCR amplification occurred in a PTC200 MJ thermocycler with the following parameters: one cycle at 94°C for 60 s, 35 cycles at 95°C for 30 s, 49°C for 55 s and 72°C for 90 s, and a final extension at 72°C for 10 min. PCR products were sequenced with the forward primer by applying the ABI 3730xl BigDye Terminator Cycle Sequencing 3.1 (Applied Biosystems) standard protocol employed by Macrogen Inc.

The phylogenetic relationships among sequences were analysed in Mega 5 [68] following a maximum likelihood approach, with bootstrap support values based on 1000 replicates, and by using as the nucleotide substitution model the GTR+G+I model determined in JModelTest 2.0.2 [69]. In addition, a Bayesian phylogenetic reconstruction was performed in MrBayes 3.1.2 [70]. In this reconstruction, four independent analyses were run considering four chains each of two million generations. Trees and parameters were sampled every 1000 generations and the default parameters were set to fit temperature and swapping. The first 25% of sampled trees were discarded as “burn-in” to ensure stabilization. The remaining trees were used to compute a consensus topology. The calyptraeid species *Crepidula plana* Say, 1822 was selected as the outgroup.

## Results

### Anatomical analysis

**Fig 2:**
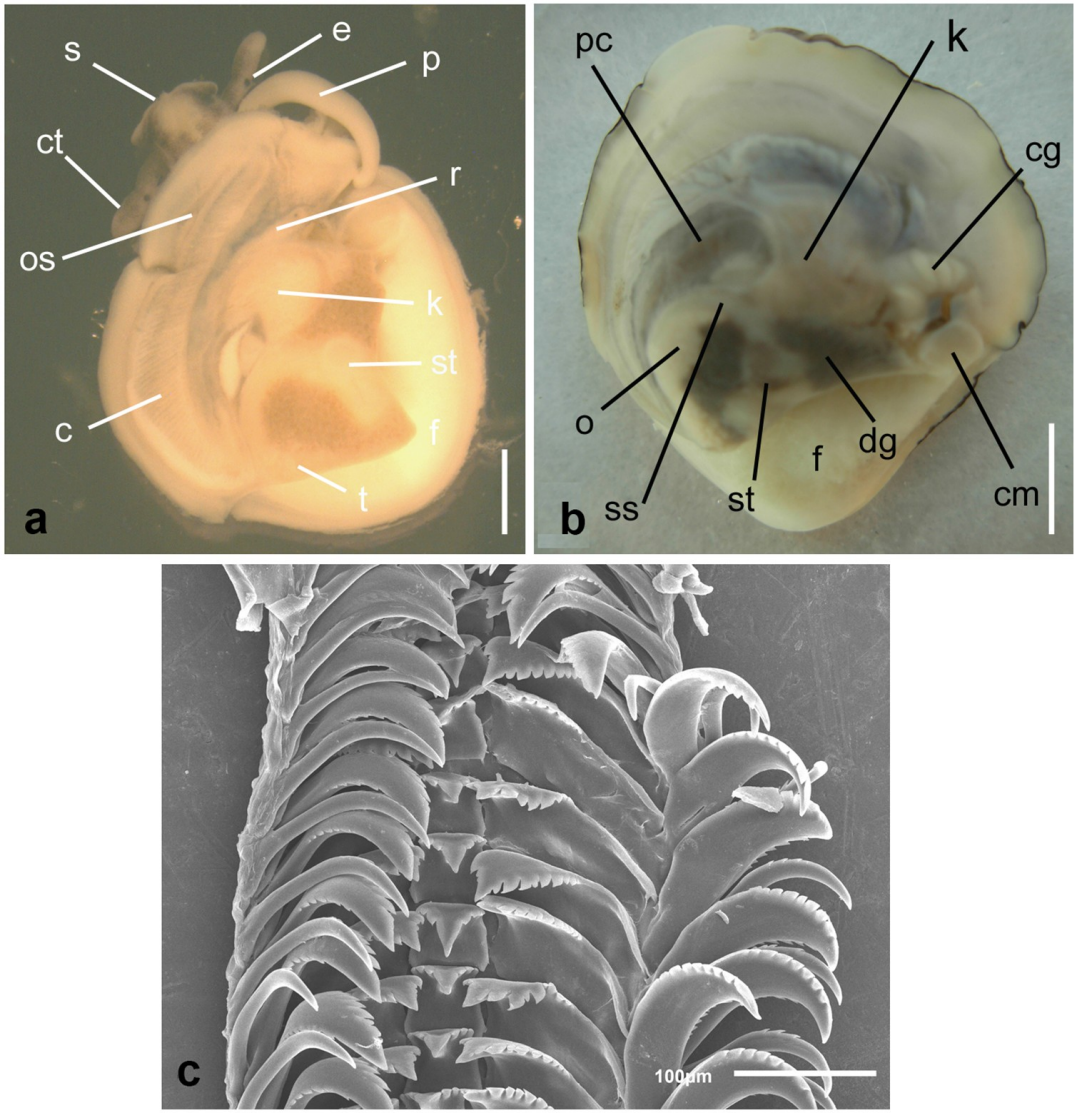
External gross anatomy and radula of *Crepipatella dilatata*. Dorsal view of a functional male (A) and a functional female (B) extracted from their shells. (C) The radula of a female from O Grove. Abbreviations: c, ctenidium; cg, capsule gland; cm, columellar muscle; ct, cephalic tentacle; dg, digestive gland; e, eye; f, foot; k, kidney; o, ovary; os, osphradium; p, penis; pc, pericarcium; r, rectum; s, snout; st, stomach; ss, style sac. Scale bars: A = 1 mm; B = 5 mm.

A detailed description of the internal anatomy, body, and shell pigmentation (S1 Appendix) and complementary figures (S1 Fig) are provided in online accessible supporting files. External gross anatomy of the head-foot and soft body pigmentation (Fig 2A and 2B) coincided with the descriptions and figures of *Crepipatella dilatata* (as *Crepidula dilatata*) provided by Gallardo [71], Collin [72] and Strebel [73]. Less apparent and finer details of the soft body such as the morphology of the male penis and female genital papilla, the arrangement of the pallial reproductive system, extension of the mantle, the dorsal arrangement of the visceral organs (Fig 2A and 2B) and the internal anatomy of the feeding system also largely match Collin’s observations [72]. Despite this, variation in certain fine anatomical traits relative to the feeding system and the osphradium, not remarked by Collin [72], and in the number of seminal receptacles are highlighted here. The osphradia of the dissected Spanish individuals were either bipectinate and asymmetric, with the external leaflets oriented towards the mantle edge being shorter than those oriented internally, or monopectinate. In most individuals, the salivary glands extended along the whole neck region reaching the nervous ring or surpassing it, but in a few individuals the salivary glands were shorter and extended only halfway along the neck region. Three to five small roundish seminal receptacles were found in brooding females.

The radulae of all the specimens from Spain here examined (Fig 2C) were typically taenioglossate, bilateral-symmetrical with a trapezoidal rachidian tooth in the centre of the radular ribbon flanked on either side by a broad lateral tooth and two slender marginal teeth, a sickle-shaped inner marginal and a thinner, hooked outer marginal. Sexual dimorphism or ontogenetical changes within the SL range of the individuals analysed were not detected. However, the morpholgy of the radulae varied slightly among individuals and within the same individual (across transversal rows). Differences among individuals and within the same individual were found in the following traits: a) the number of secondary cusps along the serrated outer edge (6–10) and along the internal edge of the lateral teeth (2–3), b) the number of secondary cusps along the inner edge (6–11) and the outer edge (5–9) of the inner marginals, and c) the number of secondary cusps along the internal edge of the outer marginal (2–5). In all specimens, the external edge of the outer marginal was smooth. Besides, despite the slight variation among and within individuals in the number of secondary cusps (3–4) of the bilateral rachidian tooth, this trait was rather conservative in non-native *C*. *dilatata*, because in most specimens (9 out of 12) the rachidian tooth presented bilaterally only three secondary cusps.

### Shell morphology

**Fig 3:**
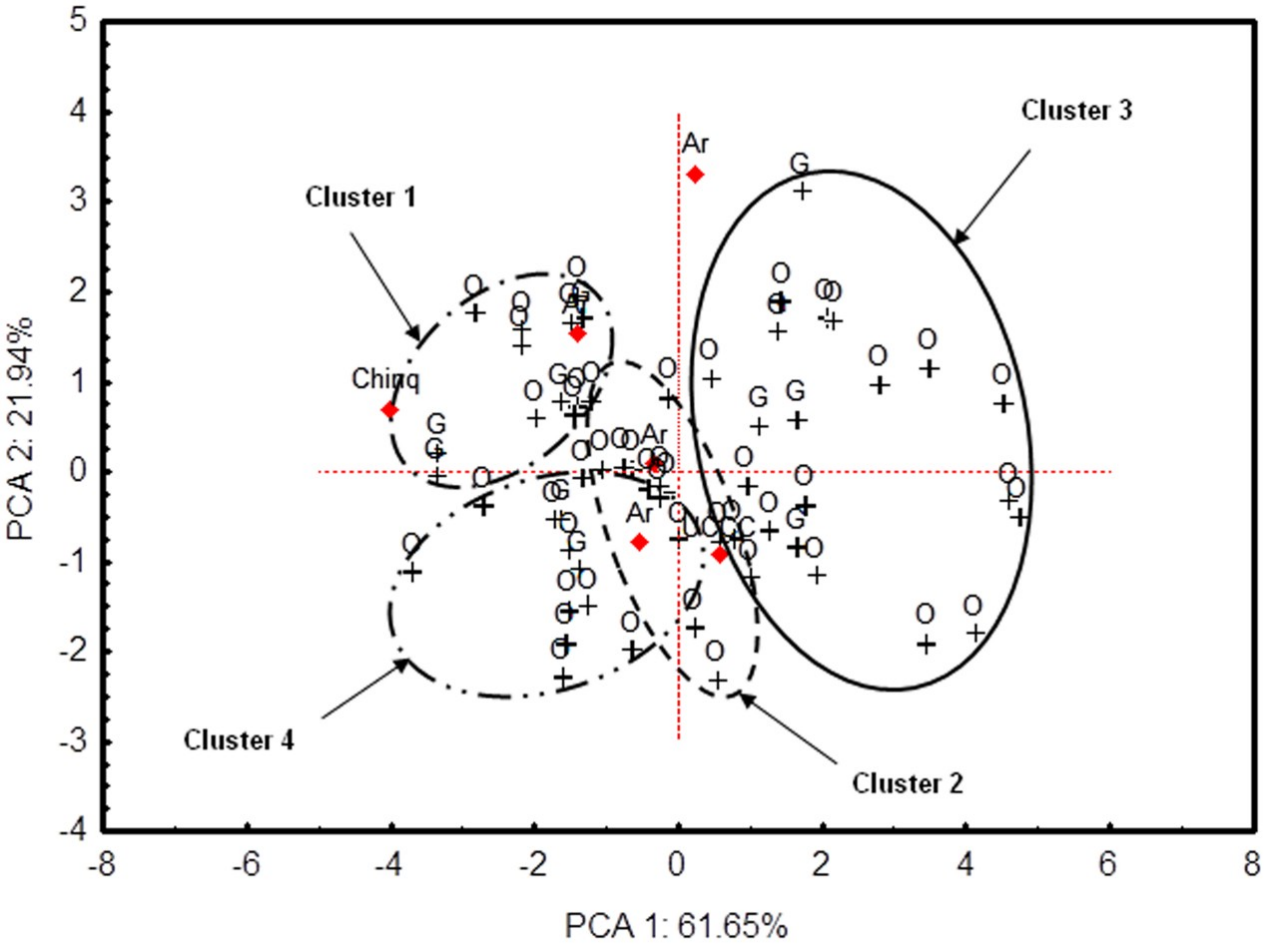
Projection of the hyperspace of morphological shell variability of non-indigenous *C*. *dilatata* onto the first two PC. Data points from a multidimensional vector space defined by seven linear and non-dimensional morphological shell variables and 59 individuals were plotted on the bi-dimensional space spanning the first two PCs of the PCA. PC 1 (= Factor 1) represented sex or overall size. Blue data points labelled with O and G correspond respectively to individuals collected from O Grove and El Arbeyal. The four ellipses with different line patterns plotted on the vector space delimit four clusters of data points obtained through the k-means clustering analysis. Each cluster represents a different shell morphotype. The cluster delimited by a continuous line includes mostly males, while the three clusters delimited by dotted lines include exclusively females. Red data points correspond to native specimens that were not used in the computation of the PCs but are projected onto the vector space for comparison.

By combining the PCA with the k-means clustering analysis using the whole dataset, the hyperspace of morphological variability was reduced to three dimensions or principal components (PCs) accumulating 98.34% of the total morphological shell variation (S1 Table) and revealing a pattern of four clusters (morphotypes) (Fig 3). Projecting the datapoints onto the bi-dimensional space defined by PC 1 and PC 2 separated clearly these four clusters from each other except for a slight overlap of cluster 2 with the remaining clusters (Fig 3).

The first principal component (PC 1), explaining 61.65% of the total variance and with high negative loadings for all of the shell variables except for SW/SL (Table 3), segregated sex fairly well (Fig 3). PC 2, accounting 21.94% of the total variance and with positive moderate loadings for SC/SS, SC/SW and SC/SL and negative moderate loadings for linear shell dimensions (SL, SW and SS), explained mainly variations in the septum (Table 3). The third principal component (PC 3), accumulating 14.76% of the total variance and highly correlated with the ratio SW/SL, which contributed most to the total variance of PC 3 (Table 3), explained mainly variation in shell aperture shape.

**Table 3:** Correlation of the shell variables with the extracted PC and variable contribution.

Variable	Factor-variable correlation (factor loadings)			Variable contribution		
	PC 1	PC 2	PC 3	PC 1	PC 2	PC 3
**SL**	-0.853901	-0.496596	0.120586	0.168971	0.160579	0.014074
**SW**	-0.868199	-0.479467	-0.090647	0.174677	0.149692	0.007953
**SS**	-0.851179	-0.498890	0.097676	0.167895	0.162066	0.009234
**SC/SS**	-0.757348	0.613699	-0.028403	0.132919	0.245240	0.000781
**SW/SL**	-0.255200	0.009228	-0.966017	0.015092	0.000055	0.903239
**SC/SL**	-0.875704	0.462302	-0.057764	0.177710	0.139166	0.003230
**SC/SW**	-0.837994	0.468958	0.252047	0.162734	0.143202	0.061489

Shell morphotypes were unevenly distributed among sexes (*Gadj* = 36.828; *P* < 10^−5^; df = 6) and among sites (*Gadj* = 8.433; *P* < 0.05; df = 3). Male shells and intersex shells were monomorphic and were found only in cluster 3 while female shells were polymorphic with 86% of the shells distributed among clusters 1, 2 and 4. Only 14% of the females belonged to cluster 3. The population in O Grove presented all of the four morphotypes with a predominance of the cluster 2 morphotype for females, while in El Arbeyal the cluster 2 morphotype was absent (Fig 3). The frequency of the different shell morphotypes did not vary among years in O Grove (*Gadj* = 4.268; *P* >0.05; df = 3).

**Fig 4:**
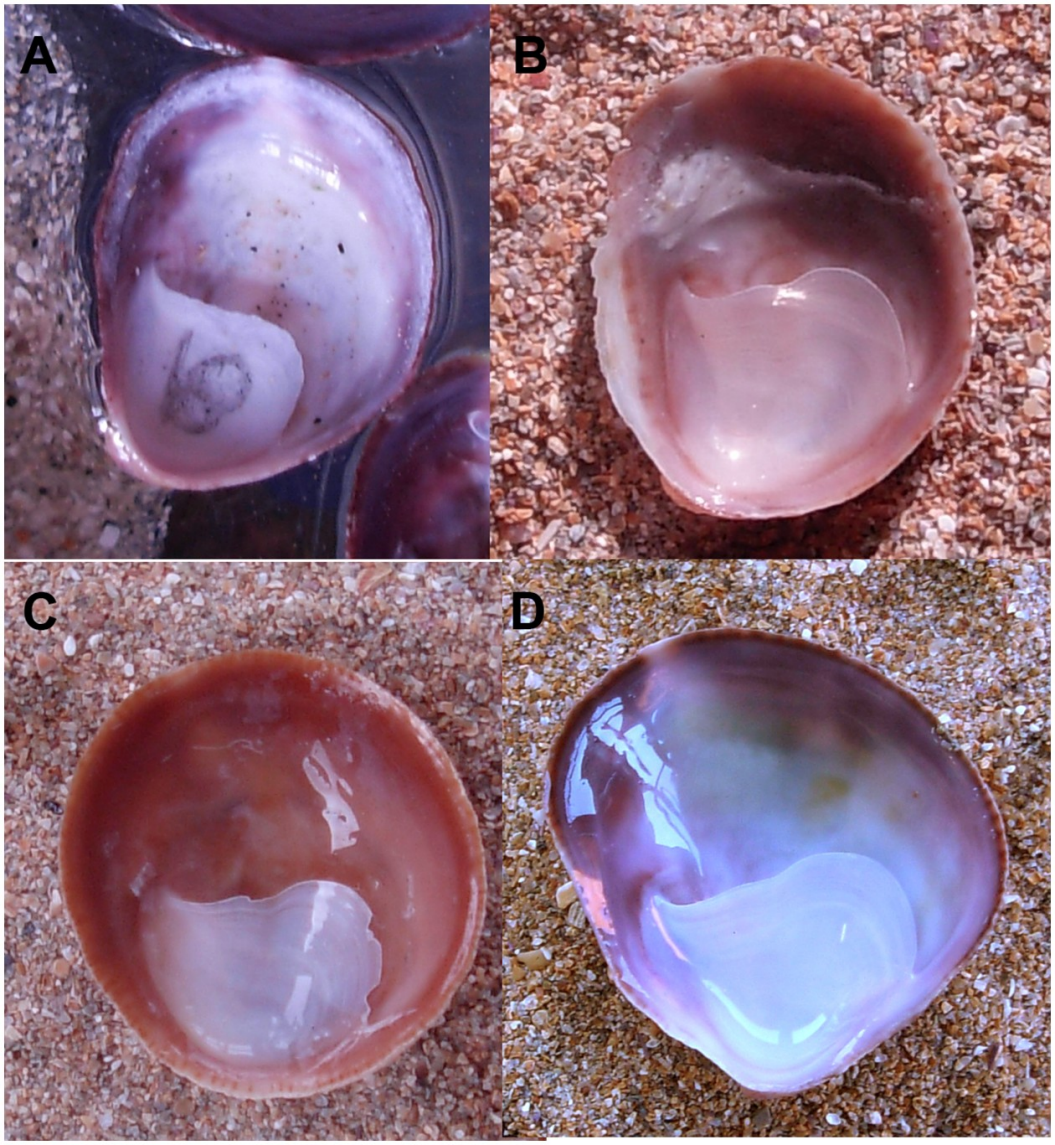
Shell morphotypes of introduced *C*. *dilatata*. Apertural view of an individual exhibiting a shell morphotype of cluster 3 (A), cluster 2 (B), cluster 1 (C) and cluster 4 (D). Scale bars: A–D = 1 cm.

Since SW/SL, SC/SS and SC/SL explained the highest variance for PC 3, PC 2 and PC 1 respectively, these explanatory variables (untransformed) were used to test for significant differences among the four morphotypes. The four shell morphotypes differed significantly with regards to their SW/SL (GLM: *F*_(3,51)_ = 3.529; P <0.05). They also differed significantly in SC/SL (GLM: *F*_(3,54)_ = 19.254; P < 10^−5^) and SC/SS (GLM: *F*_(3,54)_ = 14.727; P < 10^−5^) ratios, after removing in the latter two analyses the interaction terms, which were not significant (GLM: *F*_(3,51)_ = 0.249, P >0.05 for SC/SS; *F*_(3,51)_ = 0.611, P >0.05 for SC/SL). The unequal N HSD test revealed that females of cluster 2 (Fig 4A) had more narrow, elliptical shell apertures with the lowest SW/SL ratios that were significantly smaller than those of the other shell morphotypes (Fig 4B–4D). The other shell morphotypes had more discoidal shell apertures and larger SW/SL ratios; no significant differences were found in these morphotypes (Table 4).

**Table 4:** Post hoc comparisons of SW/SL and SC/SC ratios among the four shell morphotypes using the unequal N HSD test.

Variable	SW/SL				SC/SS			
**Mean ± SE (N)**	0.89 ± 0.048 (12)	0.80 ± 0.038 (13)	0.85 ± 0.051 (21)	0.90 ± 0.049 (13)	0.43 ± 0.014 (12)	0.33 ± 0.013 (13)	0.30 ± 0.011 (21)	0.35 ± 0.013 (13)
**Cluster**	**1**	**2**	**3**	**4**	**1**	**2**	**3**	**4**
**1**	-----------	***	**ns**	**ns**	-----------	***	***	***
**2**	***	------------	*	***	***	-------------	**ns**	**ns**
**3**	**ns**	*	------------	**ns**	***	**ns**	-----------	*
**4**	**ns**	***	**ns**	------------	***	**ns**	*	------------

Abbreviations: N = sample size; ns = non-significant differences; SE = standard error.

* P < 0.05;

*** P< 0.001

The unequal N HSD test also revealed sexual dimorphism in the SC/SL ratios. The male morphotype (cluster 3) (Fig 4B) had the lowest SC/SL ratio compared to the three female-only morphotypes (Table 4). Females of cluster 1 (Fig 4C) had the most pronounced septal curvatures with the highest SC/SL and SC/SS ratios and thus the deepest septal notches. Female shell morphs of cluster 2 (Fig 4A) and 4 (Fig 4D) did not differ significantly in SC/SL or SC/SS ratios, which were intermediate between the male morphotype and cluster 1 females (Table 5).

**Table 5:** Post hoc comparisons of SC/SL ratios among the four shell morphotypes using the unequal N HSD test.

Variable	SC/SL			
**Mean ± SE (N)**	0.25 ± 0.007 (12)	0.19 ± 0.007 (13)	0.16 ± 0.006 (21)	0.20 ± 0.007 (13)
**Cluster**	**1**	**2**	**3**	**4**
**1**	**-----------**	***	***	**
**2**	***	**-----------**	*	**ns**
**3**	***	*	**-----------**	***
**4**	**	**ns**	***	**-----------**

Abbreviations: N = sample size; ns = non-significant differences; SE = standard error.

* P < 0.05;

** P< 0.01;

*** P < 0.001

When eliminating the non-dimensional shape variables from the original dataset, the PCA combined with the k-means clustering analysis failed to recover the shell polymorphism recognized previously. The hyperspace of shell variability was reduced to a single PC 1 accumulating 97.96% of the total variance and representing sex or overal size. The datapoints were segregated into two clusters that corresponded to a “female” and a “male” morphotype as revealed by the k-means clustering analysis.

### Population traits and size at sex change

In April 2010, 47 individuals were collected from El Arbeyal; females made 68.1% of the population, males 12.7%, and intersex stages 8.5%, while juveniles represented 10.6% of the sample (S3 Dataset). The sex ratio of the population sampled was 0.19 and differed significantly from the expected 1:1 Fisherian sex ratio (χ^2^ test with Yates correction for continuity, χ^2^ = 17.82, *P* < 0.0001), with females outnumbering males 5.3 times.

**Fig 5:**
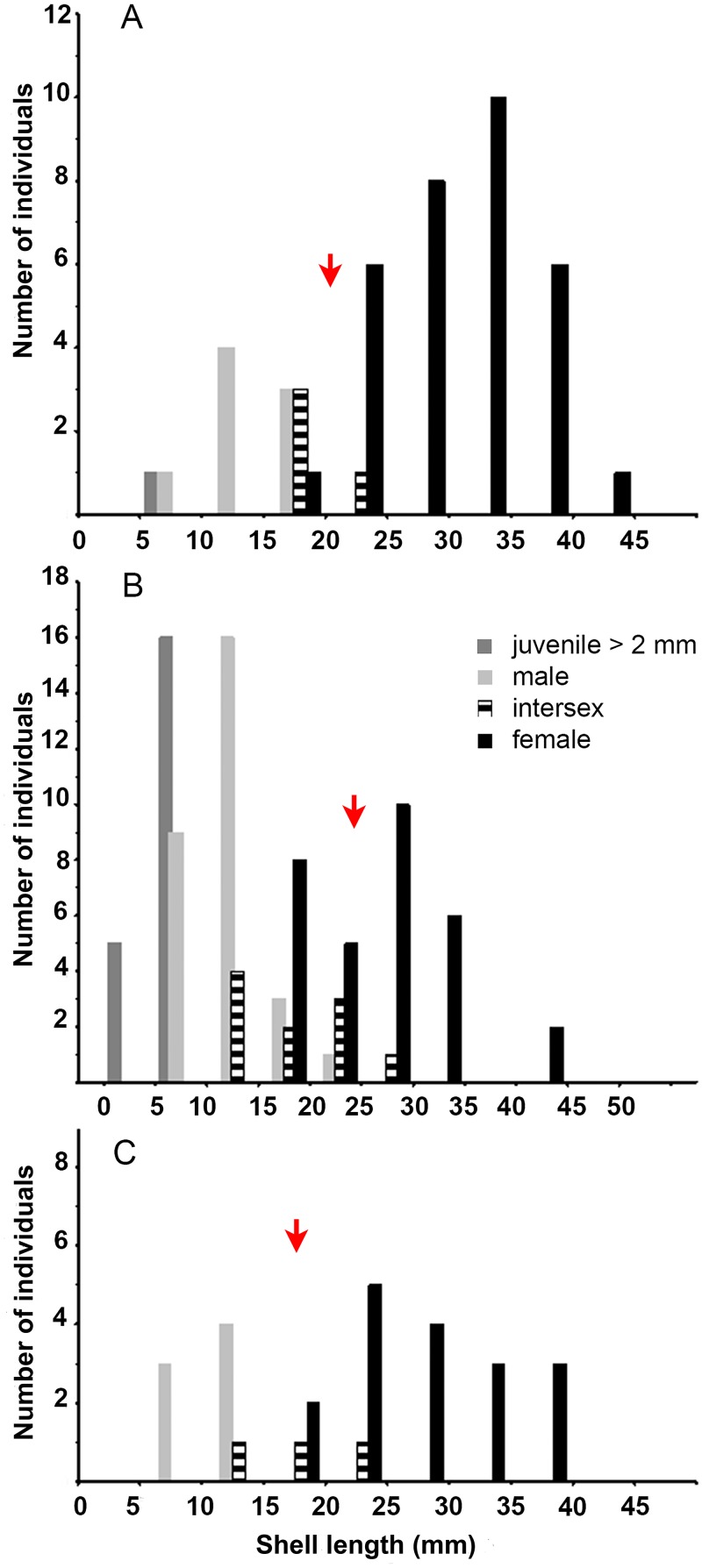
Size-gender distribution of *Crepipatella dilatata*. (A) El Arbeyal. (B) O Grove 2012. (C) O Grove 2016. Red arrow in A-C marks the estimated L_50_.

The SL of the individuals, ranging from 1.87 to 42.7 mm (mean = 24.38, SD = ± 1.58 mm, *n* = 47), was normally distributed (χ^2^ test, χ^2^ = 3.186, df = 4, *P* = 0.527) and slightly skewed to the left. Females (mean SL = 30.28, SD = ± 1.06 mm, *n* = 32; range: 18.35–42.7 mm) were significantly larger than males (mean SL = 15.07, SD = ± 1.59 mm, *n* = 6; range: 9.45–20.0 mm) (ANOVA, *F*_(1,36)_ = 35.1, *P* < 0.0001) and intersex stages (ANOVA, *F*_(1,34)_ = 33.52, *P* < 0.001) (Fig 5A). The L_50_ estimated (20.46 mm) (Fig 5A; Table 6) slightly surpassed the average SL of intersex stages (mean SL = 19.17, SD = ± 1.16 mm, *n* = 4; range: 16.5–21.9 mm) whose size distribution overlapped partially with the female and male size distributions (Fig 5A). Juveniles ranging in SL from 1.87 to 2 mm (mean SL = 1.93, SD = ± 0.061 mm, *n* = 5), representing early recruits, were still found under the protective shell of females brooding egg capsules. In October 2012, only two males (SL: 10.9 and 11.9 mm) and a single juvenile (SL = 5.48 mm) were collected on mussel aggregations growing on artificial susbtrata densely beset by the invasive cup oysters (*Crassostrea* sp.). In April 2016, the population was locally extinct.

**Table 6:** Parameters of the logistic female maturity function and estimated female L_50_.

Site and year	EL Arbeyal 2010			O Grove 2012			O Grove 2016		
		df	*P*		df	*P*		df	*P*
**L**_**50**_ **female (mm)**	20.463			24.665			17.797		
**Constant β**_**0**_	-16.248	48	*	-7.597	89	**	-9.210	25	*
**Slope β**	0.794	48	*	0.308	89	**	0.517	25	*

*P*, significance level of the fitted parameters for each population; df, degrees of freedom.

* *P* < 0.01;

** *P* < 0.001.

In contrast to El Arbeyal, the O Grove population was intensively recruiting juveniles in autumn 2012. Close to one quarter of the population were juveniles (23.1%), while 34.1% were females, 10% intersex stages, and 31.8% males (S3 Dataset). The sex ratio (0.94) did not differ significantly from 1:1 (χ^2^ test with Yates correction for continuity, χ^2^ = 0.08, *P* = 0.776).

The SL of the individuals from O Grove (mean = 16.34, SD = ± 9.15 mm, *n* = 95, range: 2.6–44.1 mm) was not normally distributed (χ^2^ test, χ^2^ = 21.2; *P*<0.001); rather it was bimodal, asymmetric, and strongly skewed to the right (Fig 5B). Females were significantly larger than males (female SL: mean = 26.38, SD = ± 1.19 mm, n = 31; range: 17.7–44.1; male SL: mean = 12.3, SD = ± 0.52 mm, n = 29, range: 8.0–20.25 mm; Mann-Whitney U test, Z = 6,53, *P* < 0.0001) and intersex (intersex SL: mean = 19.25, SD = ± 1.71 mm, n = 10, range: 14.1–29.75 mm; Mann-Whitney U test, Z = 2.99, *P*<0.01). The latter overlapped widely with the female and male size distributions. The average SL of the intersex stages was smaller than the L_50_ estimated (24.665 mm) (Table 6).

In April 2016, a total of 28 individuals which could be sexed were collected. Females represented 64% of the population sample, while males represented only 25%. The remaining three individuals were intersex stages. Recruits larger than 2 mm and outside the mother shell were absent. The population sex ratio (0.39) differed significantly from 1:1 (χ^2^ test with Yates correction for continuity, χ^2^ = 4; *P* <0.05). The population SL (mean SL = 22.04, SD = ± 0.99 mm, *n* = 28, range: 7.55–39.25 mm) was normally distributed (Chi-square test, χ^2^ = 1.567; *P* > 0.05), and females were significantly larger than males (female SL: mean SL = 27.61, SD = ± 1.59 mm, *n* = 18, range: 15.25–39.25 mm; male SL: mean SL = 10.14, SD = ± 0.67 mm; range: 7.55–12.25 mm; Mann-Whitney U test, Z = 3.81, *P* < 0.001). Female and male size distribution did not overlap. The estimated female L_50_ was 17.8 mm (Table 6) and slightly surpassed the average size of the intersex stage (mean SL = 16.42, SD = ± 3.34 mm, n = 3; range: 11.60–20.10 mm) (Fig 5C), whose size overlapped partially with the male and female size ranges. Comparisons among the different sampling periods revealed no temporal differences in female and male size in O Grove (females: ANOVA, *F*_(1,47)_ = 0.379, *P*>0.05; males: Mann-Whitney U test, Z = -1.35, *P* >0.05). However, site had a significant effect on female size, and females from El Arbeyal were significantly larger than females from O Grove (ANOVA, *F*_(1,78)_ = 5.41, *P* <0.05). Male size, by contrast, did not differ significantly among sites (Mann-Whitney U test, Z = 1.567, *P* < 0.05).

### Brood traits and reproductive success

**Fig 6:**
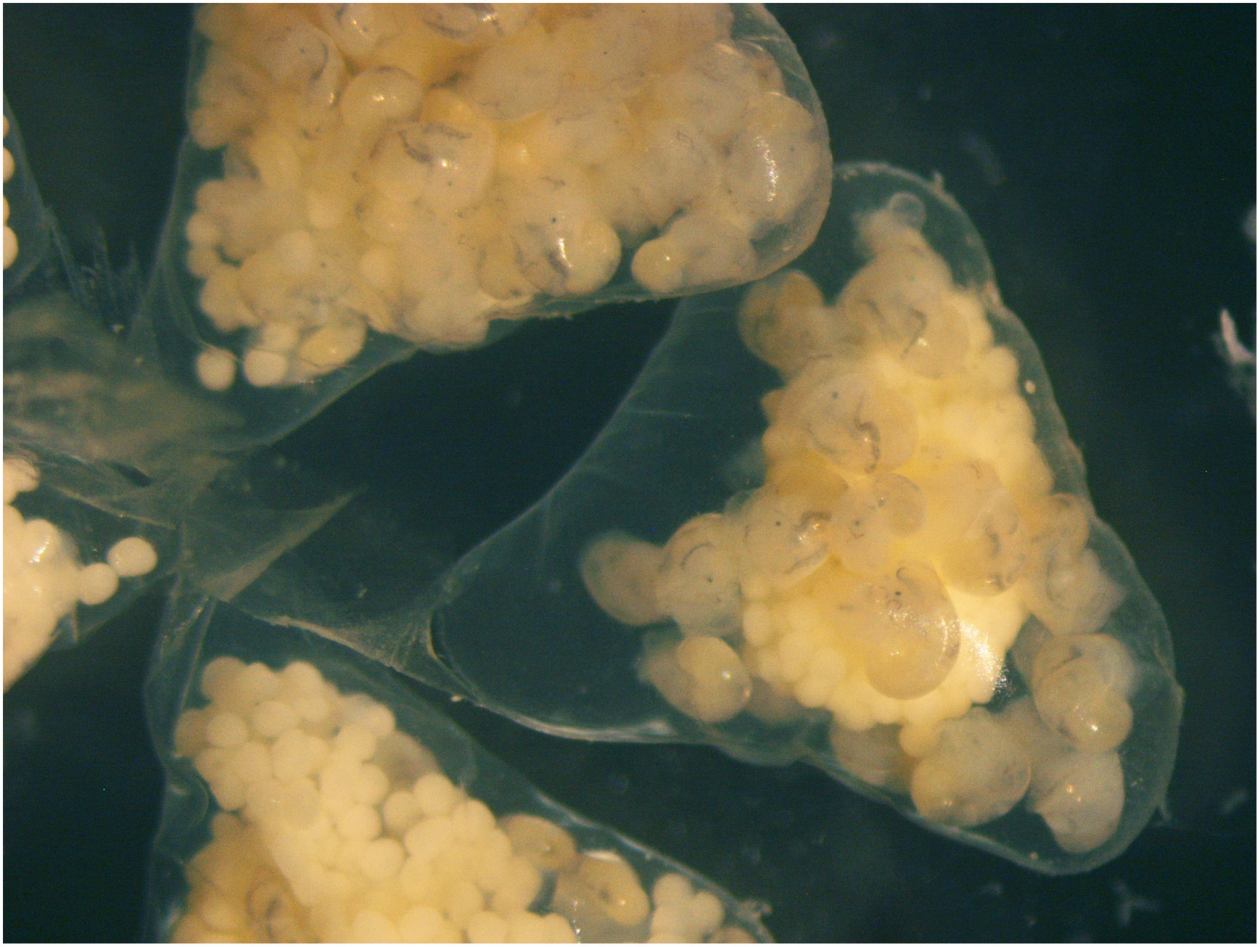
A group of stalked *Crepipatella dilatata* egg-capsules containing pediveligers and nurse eggs.

Females collected in each sampling event brooded a variable number of egg capsules. In El Arbeyal, approximately 53% of the female population was brooding, while in O Grove the proportion of brooding females in autumn 2012 and spring 2016 was 26% and 94%, respectively. The egg-capsules measured between 1.7 and 5.6 mm in maximum width (mean = 3.64, SD = ± 0.712 mm, n = 128) (S4 Dataset) and were surrounded by a transparent mucous wall, more or less triangular in outline, with a flat belt-shaped peduncle (Fig 6; S2 Fig).

Freshly laid egg capsules contained whitish to yellowish viable eggs and nurse eggs embedded in a capsular fluid, which in most broods was transparent and of low viscosity. However, a few females from O Grove collected in April 2016 endowed some of their eggs with a very thick and milky-white capsular fluid of gelatinous consistency. Older egg capsules contained larvae in advanced stages of development (early veligers or pediveligers) or juveniles surrounded by masses of nurse eggs on which they fed. In most broods with advanced stages of development, nurse eggs were uncleaved, but a few females from O Grove produced nurse eggs with anomalous cleavages. In a few egg-capsules, nurse eggs were completely depleted and only ready-to-hatch juveniles were found. In each sample, within a brood, the development of viable embryos was synchronous, but among females development was asynchronous. From some broods collected in O Grove in April 2016, offspring hatched as crawling juveniles or pediveligers (less than 10%).

**Fig 7:**
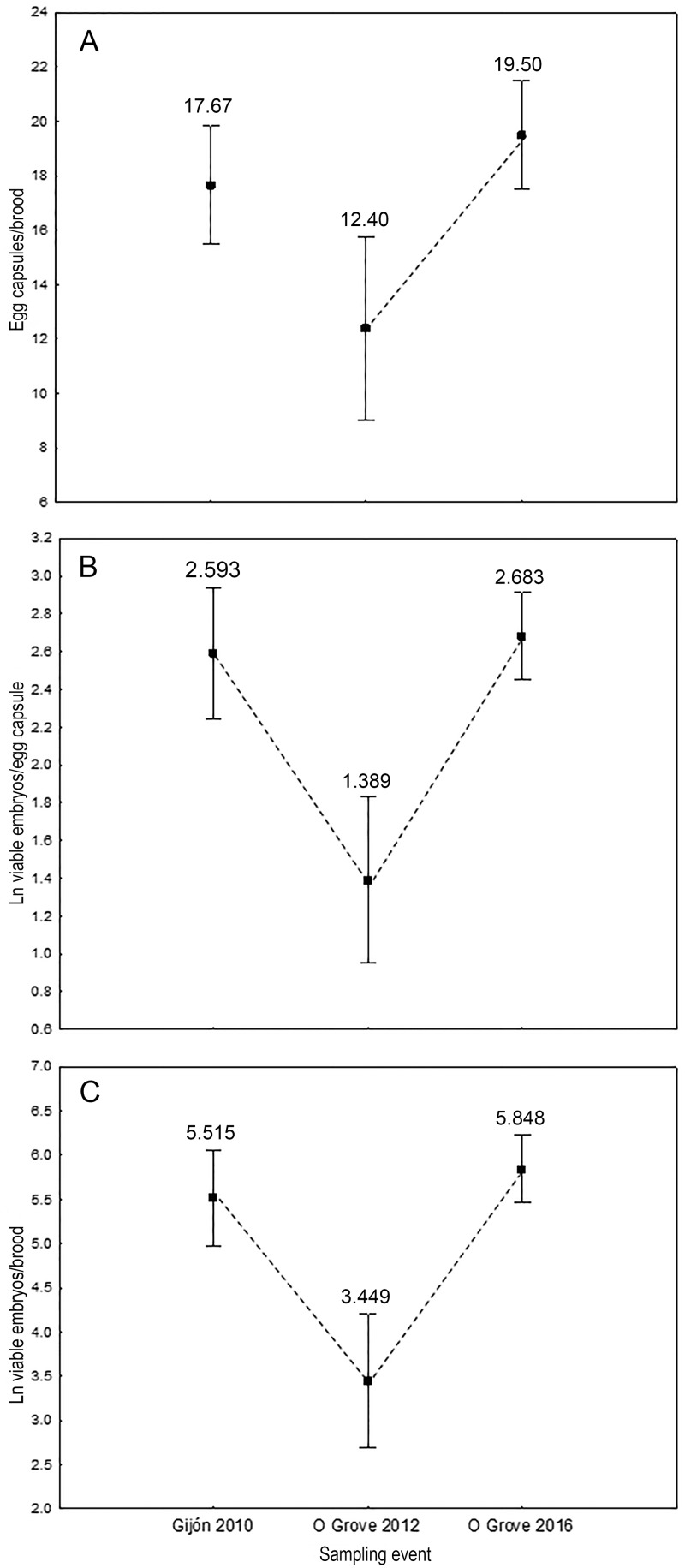
Comparison of the reproductive output among sampling events. Differences among samples in (A) the number of egg capsules per brood, (B) the number of viable embryos per brood (log-transformed) and (C) in the total number of viable embryos per brood (log-transformed) produced by females when controlling for female size. Vertical bars represent 95% confidence intervals. The small squares inside the box represent the unweighted means. Numbers above bars represent unweighted means. Discontinuous connecting lines among pairs denote significant differences at *P*<0.05 (A) and at *P*<0.01 (B, C) according to the unequal N HSD test.

Reproductive success varied greatly in introduced populations of *C*. *dilatata*. The total number of egg-capsules incubated per female across samples (5–26) co-varied with female size (*F*_(1,25)_ = 12.046; *P* < 0.01) and differed significantly among samples (*F*_(2,27)_ = 12.216; *P* < 0.001) after removing the non-significant interaction term in the hypothesis testing (*F*_(2,25)_ = 1.089; *P* > 0.05) (Fig 7A). The unequal N HSD excluded a site effect on the number of egg-capsules brooded by females and revealed significant temporal variation in broods from O Grove. Females from O Grove brooded on average significantly more egg-capsules in spring 2016 than in autumn 2012 (Fig 7A). Other pairwise comparisons did not render significant differences.

The number of viable embryos per egg-capsule (range across all broods: 2–30) and the number of viable embryos per brood (range across all broods: 18–715.5) (S5 Dataset) were also variable. Their responses to the different factors were however more complex. The total number of viable embryos per brood co-varied with female size (*F*_(1,10)_ = 7.193; *P* < 0.05) and differed significantly among samples (*F*_(2,10)_ = 23.110; *P* < 0.001) after removal of the non-significant interaction term (*F*_(2,8)_ = 2.725; *P* < 0.05). The total number of viable embryos/egg capsules, co-varied with female size (*F*_(1,25)_ = 9.296; *P* < 0.01) and differed significantly among samples (*F*_(2,25)_ = 9.839; *P* < 0.001), but there was also a significant effect of the interaction term (*F*_(2,25)_ = 10.330; *P* < 0.01). As for the number of egg capsules, the unequal N HSD revealed a seasonal effect and excluded a site effect on both the number of viable embryos/egg capsule and the total number of viable embryos/brood. Females from O Grove produced in autumn 2012 significantly fewer viable embryos per egg-capsule and per brood than females from the same site and from Gijon in spring 2016 and 2010 respectively (Fig 7B and 7C). However, the amount of viable embryos per egg-capsules and per brood that females from Gijon in 2010 and from O Grove in 2016 incubated in spring did not differ significantly (*P* > 0.05; Fig 7B and 7C).

In O Grove, the total number of eggs per egg-capsule varied between 46 and 977 (mean = 549.00, SD = ± 75.71, *n* = 15) and estimated female fecundity ranged from 962.5 to 14247 (mean = 6487.14, SD = ± 1904.44, n = 7) in September 2012. In April 2016, the number of eggs per egg capsule averaged 393.3 (SD = ± 138.81) (range: 66–686; *n* = 4) and the fecundity varied between 2712 and 16042 (mean = 9377.0, SD = ± 6665.0, *n* = 2). Thus, in O Grove, approximately 96% and 99% of the eggs incubated by females were endowed as nurse eggs for the developing embryos in April 2016 and September 2012, respectively.

### Gregariousness and social control of sex change

**Fig 8:**
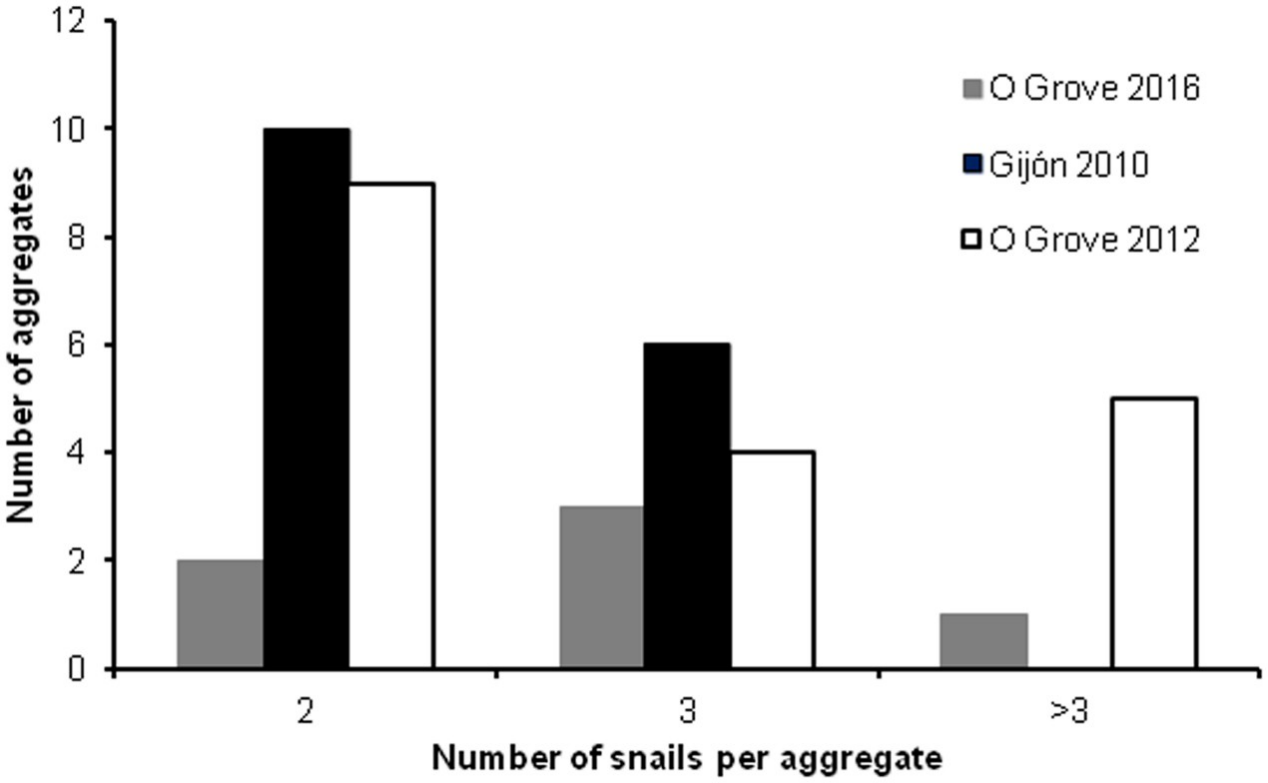
Frequency distribution of aggregate size in introduced populations of *C*. *dilatata*.

In El Arbeyal, half of the females collected were solitary and did not show any sign of home scars. The other half (SL range: 20.2–42.7 mm) exhibited one or two marked home scars on their shell surfaces that were as small as 7 mm and as large as 30.05 mm. Males and intersexual phases lacked home scars on the tops of their shells. Most home scars appeared on the right side (ten females) of the shells, the usual side where males cling, and to a lesser extent on both sides (four females) or only on the left side (two females). In six females, the home scars were arranged in two to three concentric rings of increasing diameter with the smallest, innermost rings varying from 7 to 13 mm in diameter and the largest, outermost rings ranging from 14 to 25.5 mm. The sex ratio from the aggregates averaged 0.7 (SD = ± 0.21) (*n* = 10; range: 0–2). Aggregates in which males equalled females in number predominated (50%) over aggregates exclusively formed by females (30%) or exclusively formed by males (10%). A single aggregate was formed by a female and an intersex stage (10%). The slipper limpets tended to form couples or small aggregates of three individuals with an average of 2.38 (SD = ± 0.5, *n* = 16) snails per aggregate (Fig 8).

**Fig 9:**
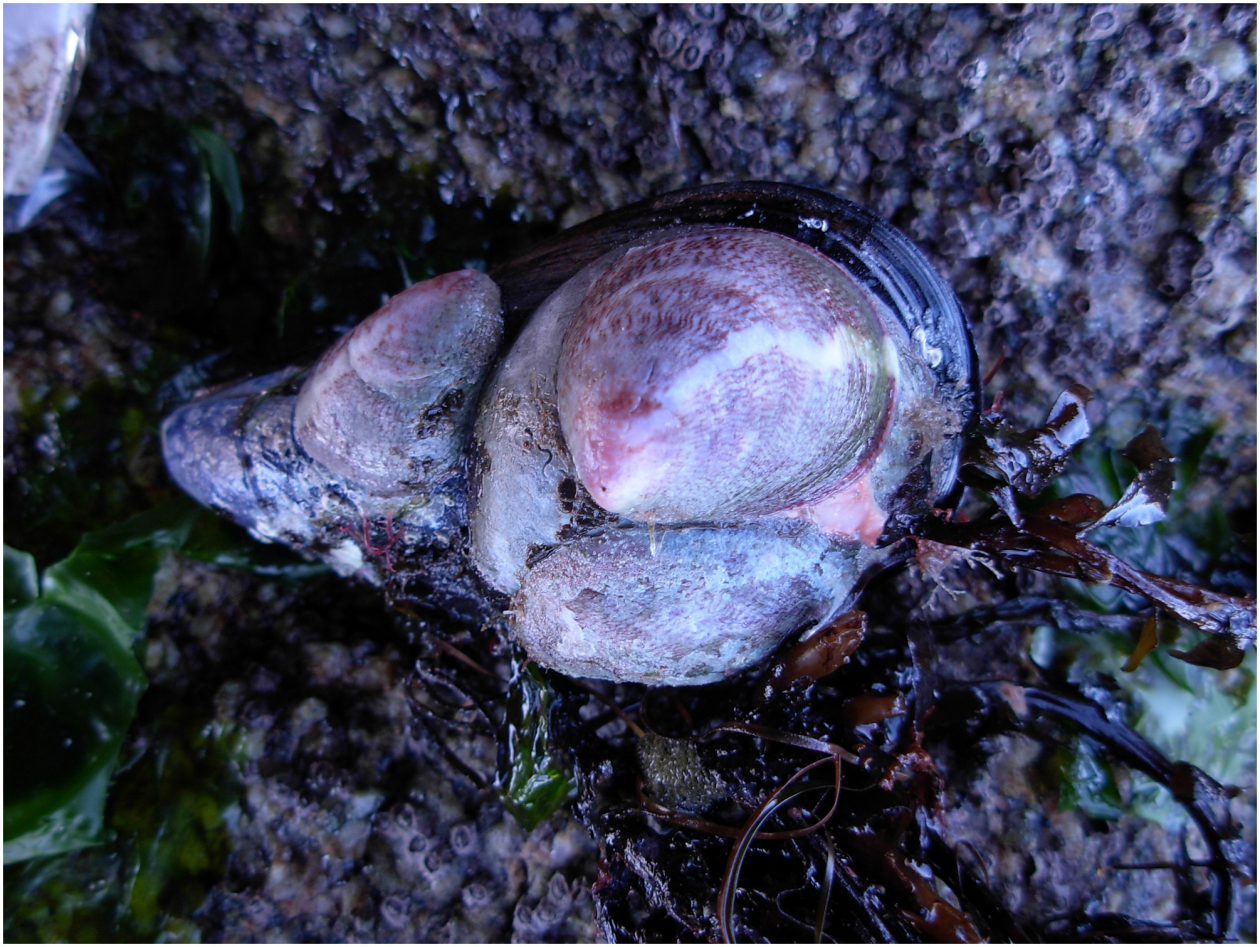
A living native mussel from O Grove with a *Crepipatella dilatata* aggregation.

In O Grove, approximately 63% of the slipper limpets aggregated into stacks in autumn 2012 and around 60% did so in spring 2016 (Fig 9). Aggregates contained up to a maximum of six members in autumn 2012 and five in spring 2016 and with respective average group sizes of 3.0 (SD = ± 1.33, *n* = 19) and 3.0 (SD = ± 1.10, *n* = 6). The frequency distribution of aggregate size did not vary temporally (*Gadj* = 1.411; *df* = 2, *P* > 0.05). In contrast, the intensity of gregariousness was site dependent. When pooling the data from O Grove, the frequency distribution of aggregate sizes was heterogeneous among sites (*Gadj* = 6.383; *df* = 2; *P* < 0.05), and large aggregate sizes tended to be more frequent in O Grove than in Gijon (Fig 8). The sex ratio of aggregates including females varied from 0 to 2 and was close to gender equality (mean = 0.89, SD = ± 0.19, *n* = 21). Males equalled females in number in slightly more than half of the aggregates (52.4%) and outnumbered females in 19% of the aggregates. Aggregates composed exclusively by females or females and intersex stages represented 23.4% of the aggregates. A single aggregate was composed of a male and an intersexual stage.

**Fig 10:**
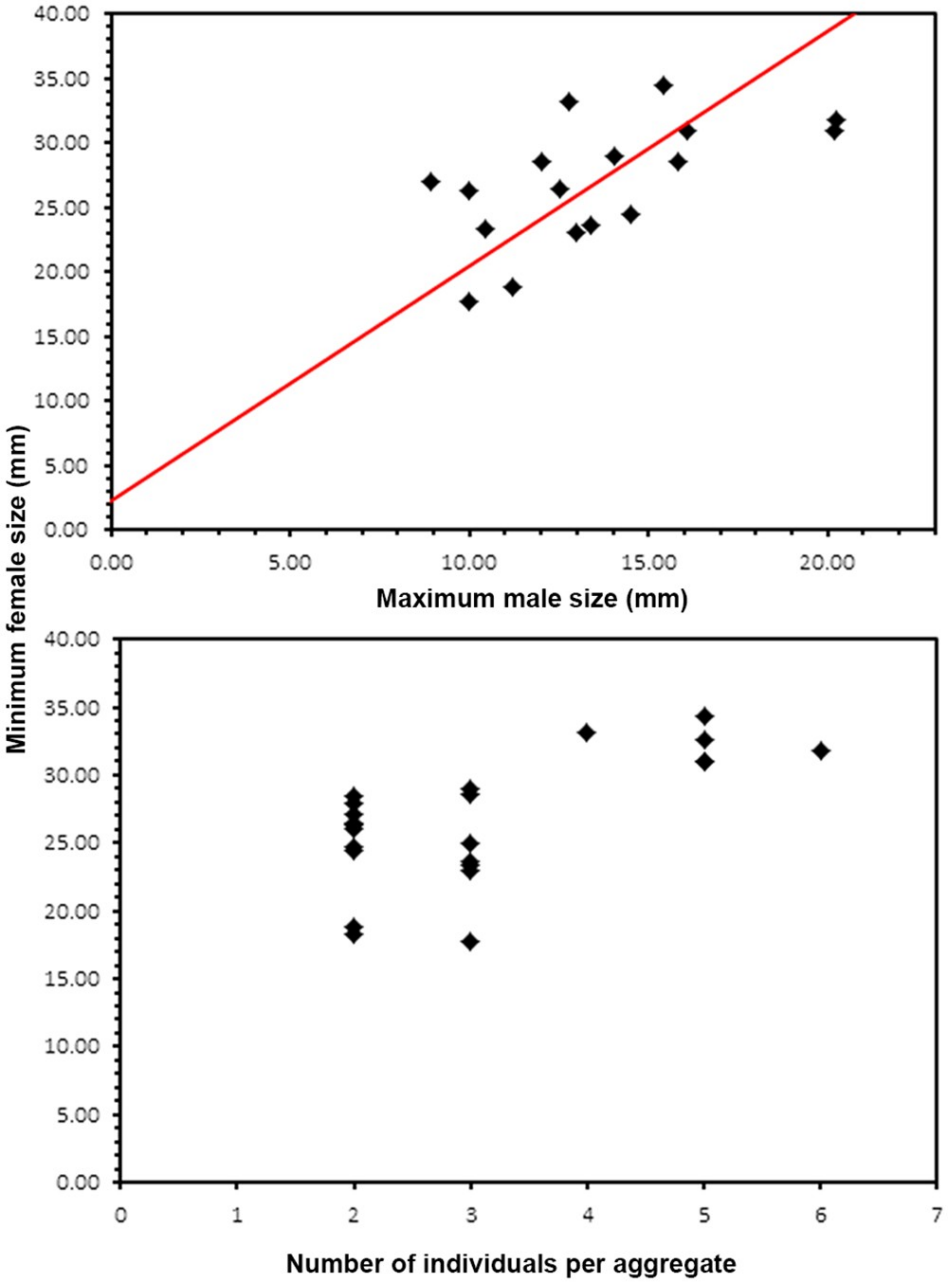
Social control of sex change in the O Grove population. (A) Functional relationship between the smallest female size and the largest male size in the aggregates. (B) Correlation between the smallest female size in the aggregate and aggregate size. Red line in (A), fitted major axis of model II regression.

In O Grove, some evidence for socially regulated sex change was detected. The SL of the smallest female in the aggregations from O Grove was positively and significantly correlated with maximum male SL (Pearson, *r* = 0.59, *F*_(1,15)_ = 7.997, *P* < 0.05) (S3 Dataset). Model II regression analysis showed that it increased significantly and linearly with male SL at a constant rate of change of 1.82 (Fig 10A). Smallest female SL was also positively and significantly correlated with aggregate size (Spearman rank correlation, *rs* = 0.56, N = 24, *P* < 0.05) (Fig 10B). By contrat, the mean SL of the members of the aggregates and aggregate size were not correlated (Spearman rank correlation *rs* = -0.035, N = 24, *P* > 0.05).

### Genetic identity and potential source population

**Fig 11:**
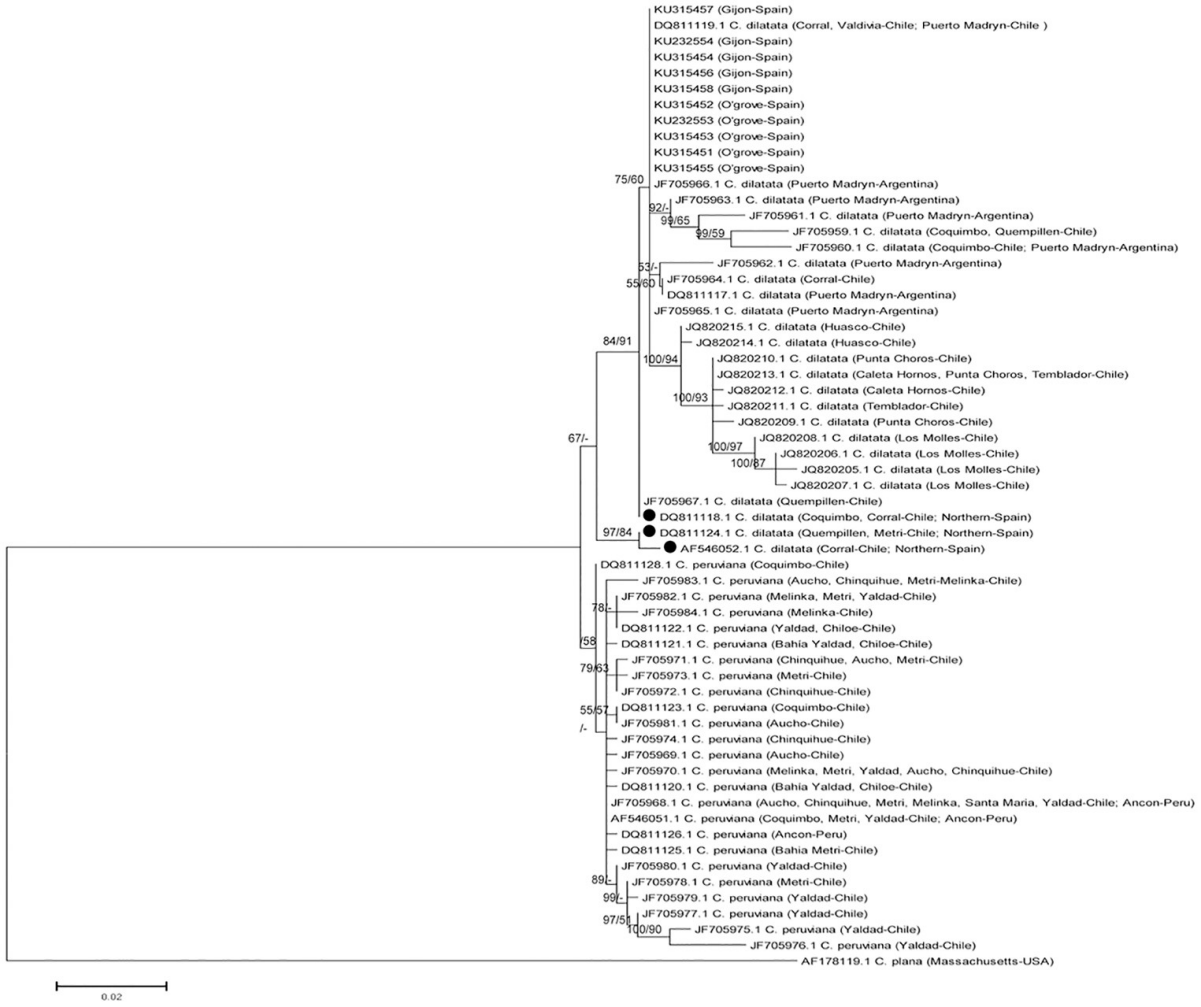
Bayesian consensus tree obtained from the analysis of the COI gene. Sampling location and the GenBank accession number are given for each sequence when available. Values above the nodes indicate *a posteriori* probabilities and bootstrap support for the MP and ML analyses, respectively.

The ten sequences from individuals from the two localities analysed were identical. Both, the maximum likelihood and Bayesian phylogenetic trees confirmed that specimens represented the species *C*. *dilatata* with a node support greater than 90% (Fig 11). Moreover, the single haplotype found was identical to a haplotype previously reported for the locality of Corral in southern Chile (DQ811119.1) and for Puerto Madryn in the south of Argentina (JF705965.1 and JF705966.1) (Fig 11). Interestingly, the haplotype found in this study was different from the haplotypes reported for northern Spain by Collin et al. [52].

## Discussion

In cryptic species complexes, the risk of overlooking a cryptic introduction is high and the use of complementary approaches for unambiguous taxonomic identification is necessary to monitor the introduction of NIS [7, 75]. In the present study, we used morphological, reproductive and molecular data to confirm the presence of the non-indigenous South American slipper limpet *C*. *dilatata* in two geographically distant populations in the Iberian Peninsula, one located in El Arbeyal, (Gijon, Central Northern Spain) and another in O Grove (Arousa Estuary, NW Spain). Genetic data and commercial statistics suggest localities from Southern Chile as the most likely source of introduction with the transoceanic fresh mussel trade serving as the pathway of introduction and live, farmed *M*. *galloprovincialis* mussels acting as vectors. In addition, we explored behavioural aspects (gregarious behaviour and socially controlled sex change) as well as population and life history traits of the populations studied to detect potential biological factors favouring the establishment and expansion of this organism in the Iberian Peninsula. Our results strongly support that the successful establishment and spread of *C*. *dilatata* in Spain has been favored by a combination of plastic responses and characteristic life history traits, such as the direct embryonic development, the gregarious behaviour exhibited by adults, and the plasticity observed in body size, female maturity, timing of sex change, reproductive investment and sex ratio.

### Taxonomic identification and morphological variability

The slipper limpet *C*. *dilatata* together with *C*. *peruviana* and *C*. *occulta* forms a cryptic species complex in the native range of *C*. *dilatata* [33, 37, 71, 76]. The genetic evidence provided here, i.e. COI gene sequences, confirm the presence of *C*. *dilatata* in the Spanish localities of El Arbeyal, at Gijon, and in O Grove. In addition, anatomical characteristics of the specimens collected in El Arbeyal and O Grove showed close resemblance to the internal and external gross anatomy of native *C*. *dilatata* described by Gallardo [71], Collin [72] and Strebel [73]. The radular teeth and the penis morphology allowed us to discriminate the cryptic species *C*. *dilatata* and *C*. *occulta*. Contrary to *C*. *occulta* [40], the penises of the *C*. *dilatata* individuals examined here lacked a penial papilla like native ones [72]. The radulae of the non-indigenous *C*. *dilatata* individuals did not show sexual dimorphism but varied among and within individuals for some traits with some radulae matching with those from native specimens from Valparaiso studied by Troschel [74]. Despite this variation in non-native *C*. *dilatata* radulae, differences with those of *C*. *occulta* were found in the number of secondary cusps in the rachidian and in the non-overlapping ranges of the number of secondary cusps in the inner marginals; non-native *C*. *dilatata* radulae had three secondary cusps in the rachidian, by contrast *C*. *occulta* had 2–5 [40]. Besides, the inner marginals of the former species had 6–11 and 5–9 secondary cusps respectively in the inner and outer edge, while in *C*. *occulta* the corresponding ranges were 5–12 and 3–6 [40]. Although Veliz et al. [37] reported only two secondary cusps on each side of the central tooth for native *C*. *dilatata* from La Herradura Bay (Northern Chile), the third and most basal secondary cusps are quite reduced and could be easily overlooked. Alternatively, it may be possible that in *C*. *dilatata* the central tooth is ecophenotypically plastic or the species is polymorphic for this trait; i.e. differences in morphology could be correlated with ecological or geographical features that confer adaptation to different selective regimes. Intraspecific, non-ontogenetic and non-sexual differences in the radula have been previously reported in other marine gastropods [77, 78] including the invasive slipper limpet *C*. *fornicata* [35].

Some traits that are taxonomically informative for Calyptraeidae, such as the morphology of the osphradium and salivary glands [72], proved to be more variable in the non-native *C*. *dilatata* collected in this study than in the native specimens studied by Collin [72]. The osphradium in non-native specimens was bipectinate and asymmetric or monopectinate, and the salivary glands reached the nervous ring or were shorter and extended only halfway along the neck region. In native *C*. *dilatata* specimens, the salivary glands run along the whole neck length, and the osphradium is monopectinate [72]. The variation in anatomical traits observed in non-native *C*. *dilatata* compared to that of native populations deserves further study to elucidate potentially plastic responses or local adaptation in this species.

Spanish specimens of *C*. *dilatata* were also polymorphic in shell shape. Part of this morphological variation could be explained by sexual dimorphism. Males were monomorphic and had a morphotype (cluster 3) that was clearly different from a group of three morphotypes found only in females. This male morphotype differed from the female shell morphotypes by having a lower SC/SL ratio. Additionally, Gallardo [71] reported ontogenetical differences in the shape of the septum edge that was correlated with size in native *C*. *dilatata*. Further evidence indicates that changes in the SC/SL ratio may parallel changes in the reproductive function of the septum or associated organs. Larger SC/SL ratios, as found in females here, imply a deeper notch in the left side of the septum and a left sided extension of the mantle edge along the free edge of the septum. Taking into account that *C*. *dilatata* is a protandric species and that females incubate their offspring in the free space between the free edge of the septum and the mantle edge [31], an increase in the SC/SL ratio during ontogeny allows for an expansion of the brood area per unit of shell aperture area in individuals changing sex from male to female. Sexual dimorphism in shell shape also occurs in other gastropod species with separate sexes [16, 79, 80]. For example, females of the boreal species *Margarites vorticiferus*, which brood their egg masses inside the umbilicus, have a significantly larger umbilicus than males [79].

An interesting result of the present study was the shell shape polymorphism in Spanish females and the uneven distribution of shell morphs among sites. O Grove exhibited the complete set of shell morphs, while a female shell morph with the lowest SW/SL ratio (cluster 2) was absent in El Arbeyal. Gallardo [71] has documented shell aperture shapes ranging from ovoid to disc-shaped for sessile *C*. *dilatata* specimens in their native range. In addition, native specimens from Argentina and Chile (S1 Dataset; Fig 3) exhibit also the three female morphotypes revealed in our analyses. Yet, the causes for females being polymorphic and the differences in the distribution of female shell morphs among sites in the introduced range are unclear.

In non-native *C*. *dilatata*, the shell morph with the lowest SW/SL ratio (cluster 2) tended to be more convex than the other shell morphs [Fig 9; A. Richter pers. obs.]. In Calyptraeidae, shell convexity is correlated with the capacity to form stacks [35]. Stack-forming species like *C*. *onyx* and *C*. *fornicata* are more convex than species that do not form chains [35]. Among stack-forming species, shell morphs that are highly convex and narrow form larger chains than shell morphs that are broader and shallower [81]. In Spanish specimens of *C*. *dilatata*, a maximum of five to six members per stack was observed in O Grove, while in El Arbeyal, where the cluster 2 shell morph was absent, stacks were formed by a maximum of three individuals. This variability in gregarious behaviour may contribute to the polymorphic shapes observed in the present study. Environmental factors such as wave exposure and predation risk may also account for the females being polymorphic and the polymorphism in shell shape among sites. In intertidal gastropods, these factors have been reported as determinants of shell shape [78, 82–84], and in Calyptreaidae, it has been suggested that shell convexity influences the vulnerability to predation by shore crabs. [85]. Yet, whether the polymorphism in female shape observed in non-native *C*. *dilatata* is due to plastic responses to environmental factors, to local adaptation, or to a combination of both remains to be unravelled.

### Social behaviour, reproductive strategy and invasion success

Gregariousness and hermaphroditism are behavioural and reproductive strategies that may favor invasion by non-native species mainly by maximizing successful mating and by avoiding or mitigating Allee effects [11]. In fact, when considering marine benthic fauna, many non-native polychaetes, cup-oysters, shipworms, dwarf mussels, gastropods and ascidians that have successfully established outside their native ranges are either gregarious, hermaphroditic, or both [4, 15, 44, 92–95]. Gregariousness may occur throughout most of the benthic life phase or be restricted to the breeding season, as in the invasive Japanese oyster drills *Ocinebrellus inornatus* and *Rapana venosa* [92]. Within Calyptraeidae, whose members are all protandric, NIS that have successfully established and spread are *C*. *fornicata* and *C*. *onyx* [29, 44, 96]. Both species exhibit permanent gregarious behaviour and sex change is socially controlled [15, 17, 20, 81]. Particularly, the invasive success of *C*. *fornicata* has been explicitly linked to its gregariousness and socially regulated sex change, since gregariousness increases multiple paternity level which in turn increases intrabrood genetic diversity and improves offspring survival [15, 97]. Other calyptraeids with less plastic sexual and social behaviour have a reduced capacity for invasion. For example, *Calyptraea chinensis*, which was first recorded in 1940 in Oosterschelde, Netherlands [41], and *Bostrycapulus odites*, formerly identified as *B*. *aculeatus* [43] and which has been introduced to the Port of Alicante, in the SE coast of Spain in the 1970s [42], have become extinct or are locally very restricted. Both species form small, brief aggregations of a maximum of three individuals [38, 39, 98]. In *C*. *chinensis*, sex change does not depend on social interactions [38]. This may hold true for *B*. *odites*, because calyptraeids that are minimally gregarious or solitary generally have a very low or no sexual response to social interactions [17, 38, 81].

Although it is clear that invasion success depends on various factors, the contrasting establishment success among slipper limpets differing in their intensity of sociality and in their plastic sexual response to social cues, suggests that within this family strong social behaviour and socially regulated sex change might be determinant for invasion. Our results lend some support for this hypothesis. As revealed by our study, non-native populations of *C*. *dilatata* were gregarious and tended to form semi-permanent to permanent stacks that lasted enough time to allow for the growth and sex change of male hitchhikers. The presence of concentric home scars on the left and right side of the shells of focal females and the existence of multi-female stacks further supported this notion. However, the intensity of gregariousness varied significantly among sites. In O Grove, where the population was successfully established, sociality was more intense than in El Arbeyal, where it experienced a demographic decline through time until complete extinction; in O Grove, up to 5–6 individuals aggregated while only three individuals aggregated in El Arbeyal, and aggregates with more than three members were significantly more frequent in O Grove than in El Arbeyal. In addition, in O Grove, gregariousness influenced the sexual behaviour of stack members. The smallest female SL in the aggregate was positively correlated with the largest male SL and tended to be twice as large as the largest male SL. Minimum female SL was also positively correlated with aggregate size, while there was no correlation between mean SL of the individuals in the aggregate and aggregate size. Together these facts strongly indicated that, in O Grove, members of each aggregate can adjust their size at sex change to local mating conditions, showing high plastic responsiveness to social interactions in the timing of sex change. A relationship between aggregate composition and timing or size at sex change has been reported in other protandric marine gastropods with socially controlled sex change; this includes calyptraeids [17–21, 64].

Theoretical models predict male-biased sex ratios for protandric species [99], however protandric species can also have balanced sex ratios or female biased sex ratios [15, 17, 20, 99]. The sex ratio of the non-native Spanish populations tended to be more balanced and less female biased than that of the native populations where it was strongly female-biased (Table 7). In O Grove, the sex ratio shifted from a balanced sex ratio in autumn 2012 to a slightly female-biased sex ratio in spring 2016, while in El Arbeyal, the sex ratio was female-biased in April 2010. A female-biased sex ratio for protandric species may indicate that the population is aging and recruitment is low, since females are in the largest size group and thus oldest age classes [17]. In El Arbeyal, low recruitment may have led to the strongly female-biased sex ratio in April 2012. At this site, juveniles and small males were indeed extremely scarce and the population size decreased in a time span of four years until complete local extinction in April 2016. A female-biased sex ratio may also arise due to sex-specific mortality with males suffering higher predation or wave impact. Males of *C*. *dilatata* have a more flexible and less adhesive foot [100] and may be more easily detached from the substratum than females and thus more vulnerable to wave action and predators. This could explain the sex ratio shift observed in O Grove.

**Table 7:** Reproductive traits of *Crepipatella dilatata* across its current distribution range.

Source	Site	Status	Capsules/brood X ± SD (min–max)	Eggs/capsule X ± SD (min–max)	Viable embryos/capsule X ± SD (min–max)	Viable embryos/brood X ± SD (min–max)	Fecundity X ± SD (min–max)	Nurse eggs/embryo	Female SL (mm) X ± SD (min–max)	L_50_ (mm)	Aggregate size (min-max)
**Present study**	**O Grove September 2012**	non-native	**11.1 ± 5.44** **(5–20)**	**712.4 ± 177.35** **(492–945)**	**6.0 ± 2.25** **(2–13)**	**44.9 ± 49.43** **(18–71.5)**	**8599.6 ± 4304.57** **(4949–14247)**	**117.73**	**26.38 ± 6.63** **(17.7–44.1)**	**24.67**	**1–6**
**Present study**	**O Grove April 2016**	non-native	**19.5 ± 5.02** **(10–27)**	**393.3 ± 277.61** **(66–686)**	**17.3 ± 8.7** **(3–30)**	**400.9 ± 217.98** **(136–715.5)**	**9377.0 ± 9425.7** **(2712–16042)**	**21.73**	**27.61±1.37** **(15.25–39.25)**	**17.80**	**1–5**
**Present study**	**El Arbeyal, Gijón, April 2010**	non-native	**17.0 ± 3.92** **(9–23)**	**?**	**14.4 ± 1.7** **(6–22)**	**247.08 ± 40.36** **(81–396)**	**?**	**?**	**30.28 ± 6.0** **(18.35–42.7)**	**20.46**	**1–3**
[52]	**Pontevedra Estuary**	non-native	**?**	**?**	**?**	**?**	**?**	**?**	**(?- 36)**	**?**	**?**
	**Ŷ; average CV**	non-native	**15.87; 27.18%**	**552.85; 40.81%**	**12.57; 46.7%**	**230.96; 77.3%**	**8988.30; 6.12%**	**69.73; 97.35%**	**28.08; 7.04%**	**20.97; 16.51%**	**-**
[37]	**La Herradura**	native	**12.8 ± 5.7** **(4–24)**	**304.9 ± 90.4** **(223–363)**	**9.7 ± 3.1** **(1–15)**	124.16*	3952*	30.43*	**25.98 ± 6.27** **(18.8–37.0)**		**?**
[33]	**Chinquihue**	native	**25.1 ± ?** **(14–30)**	**?**	**20.39 ± ?**	**439.92**	**?**	17*	**35.13 ± ?** **(20–50)**	**?**	**1**
[33]	**Isla Tabón**	native	**?**	**?**	**?**	**?**	**?**	**?**	**?**	**?**	**1–3**
[32]	**Concepción Bay**	native	**?**	**?**	**?**	**?**	**?**	**?**	**30.98**^5^ **(29–36)**	**?**	**?**
[32]	**Mehuin 1976**	native	**14.2**^1^ **(7–20)**	**?**	**14.15**^1^	**211.76**^1^	**?**	**?**	**20.65**^5^ **(12–28)**	**?**	**1**
[86]	**Mehuin 2005**	native	**14**^2^ **(4–12)**	**94.63**^3^	**?**	**?**	**1324.8**^4^	**?**	**17.45 ± 2.27** **(12.7–20.1)**	**?**	**?**
[86]	**Quempillén 2003**	native	**10**^2^ **(3–17)**	**326.50**^3^	**?**	**?**	**3265.0**^4^	**?**	**28.85 ± 3.79** **(19.4–34.5)**	**?**	**?**
[86]	**Quempillén 2004**	native	**16**^2^ **(4–24)**	**433.627**^3^	**?**	**?**	**6938.08**^4^	**?**	**28.98 ± 2.74** **(29.7–34.4)**	**?**	**?**
[106, 107]	**Quempillén 1989**	native	**14.7**	**428.46**^1^	16.6* (15.04–18.7)	244.02*	**5485.64**^1^	**24.81**^4^ **(8–38)**	**26.52**^5^ **(18–36.2)**	**?**	**?**
[34]	**Chubut, Santa Cruz**	native	**(9–22)**	**303 ± 54** **(203–375)**	7.2* (2–12)	**?**	**?**	41.08*	**22 ± ?** **(11–32)**	**?**	**?**
[39]	**Population 1**	native	**?**	**?**	**?**	**?**	**?**	**?**	**19.03**^5^ **(10.4–25.7)**	**10.8**	**?**
[39]	**Population 2**	native	**?**	**?**	**?**	**?**	**?**	**?**	**23.95**^5^ **(14.4–33.6)**	**14.4**	**1–2**
[39]	**Population 3**	native	**?**	**?**	**?**	**?**	**?**	**?**	**25.11**^5^ **(15.0–35.8)**	**18.4**	**1–6**
	**Ŷ; average CV**	native	**15.26; 30.99%**	**315.19; 39.09%**	**13.61; 38.81%**	**254.97; 52.38%**	**4193.10; 51.08%**	**28.33; 35.75%**	**25.38; 20.29%**	**14.53; 26.15%**	

Abbreviations: CV, coefficient of variation; min, minimum value; max, maximum value; SD, standard deviation; X, sample means; Ŷ, means of means; -, non applicable; ?, no data.

* value calculated here with the data published by the authors;

^1^, value calculated here from the published regression equation and by using a here estimated mean female size;

^2^, value obtained by extrapolation from published graphs;

^3^, value calculated here from published regression equations;

^4^, value here estimated;

^5^, mean female size here calculated from a here fitted regression line using the data from the table (S3 Fig).

It is noteworthy that the social behaviour of non-native populations of *C*. *dilatata*, at least of the population from O Grove, contrasts with the sociality of *C*. *dilatata* in its native range, where in most populations, individuals tend to be solitary or moderately gregarious forming small aggregates with up to three individuals (Table 7). Additionally, others have shown that the proportion of individuals in native populations that form aggregations is also generally low (0%–29%) [32, 33, 39]. To date, plastic socially regulated sex change in native *C*. *dilatata* has never been reported, and it is not expected in native populations with solitary individuals or aggregations of 2–3 individuals per stack. While it remains to be tested whether native populations with large aggregation sizes present plastic sex change regulated by social interactions, the present results suggest that both an intensification of gregarious behaviour and socially controlled sex change in non-native *C*. *dilatata* may favour its successful establishment. Shifts in the social behaviour of successfully established non-native populations have been previously reported in social insects [24, 25]. It has been proposed that mate-finding Allee effects might be a driver for the evolution of behavioural and reproductive traits that can mitigate and/or help avoid these effects. In conditions of high population density as usually occurs in native populations, behavioural responses that mitigate Allee effects would only marginally improve fitness but would be highly advantageous in non-native populations passing through population bottlenecks [11]. Non-native populations of *C*. *dilatata* offer an opportunity to test this, since these organisms have low dispersal capabilities and asynchronous reproduction. Additionally, Gascoigne et al. [11] have suggested that populations with spatially and temporally variable sex ratios are prone to Allee effects.

Plasticity in life history traits is considered as relevant for the invasive success of NIS [12, 14]. Indeed, spatial and temporal variation in life history traits is common in invasive fauna [13, 20, 101, 102]. According to our results and those of others, *C*. *dilatata* presents plastic life history traits (Table 7). Mean female SL, size at female maturity or sex change (L_50_), and maximum SL (*Lmax*) vary greatly across the current distribution ranges (both native and introduced) of this species with values for Spanish populations falling within the range of variation observed in native populations (Table 7). Yet, compared to native populations, Spanish females showed a trend of maturing later and reaching larger SL and *Lmax*. For the non-native populations, the average value of each one of these parameters surpassed that of native populations, with the average coefficient of variation (CV) of non-native populations being much lower than the corresponding CV of native populations (Table 7). This suggests that in *C*. *dilatata*, later maturation and larger female size could be adaptive during the invasion process.

The reproductive success and parental investment of *C*. *dilatata* was also highly variable across its current geographic distribution (Table 7). Also, the variation of these reproductive traits differed among non-native and native populations. The number of egg-capsules per brood and fecundity of non-native populations varied less than in native populations. By contrast, the number of eggs per egg-capsule, the number of viable embryos per brood and per egg-capsules and parental investment tended to be more variable in non-native populations than in native ones (Table 7). In addition, parental investment of non-native populations is higher than in native populations. In marine gastropods, the amount of extra-embryonic food resources available to the encapsulated embryos has an important influence on hatching size, offspring survival, and performance (growth rate) [103–105]. Because in *C*. *dilatata* hatching size is positively correlated with the ratio of nurse eggs per embryo [106], endowing a variable amount of nurse eggs to the developing embryo is an effective mechanism for controlling hatch size and thus offspring fitness in a wide range of habitats and food availability conditions. As such, an increase both in the number of nurse eggs per embryo and in the plasticity of this trait may have contributed to the invasive success of *C*. *dilatata* in Spain.

### Source, vectors and spread of the invasion

As demonstrated by DNA barcoding, the COI gene sequences of all ten specimens collected from O Grove and Gijon were identical. The single haplotype recovered matched a haplotype of native *C*. *dilatata* present in Corral Bay (Southern Chile) (DQ811119.1) and Puerto Madryn (Southern Argentina) (JF705965.1 and JF705966.1) and was different from the three haplotypes found in the introduced populations from Beluso Bay (NW Spain) sampled in 2009 by Collin et al. [52]. In the native distribution range, one of the introduced haplotype present in Beluso Bay is restricted to the localities of Quempillen and Metri (Southern Chile). According to Geller et al. [7], the existence of different NIS haplotypes that have non-overlapping distributions in the native range is strong evidence for multiple introductions. Based on this, our results suggest that the introduced populations of *C*. *dilatata* in the Atlantic coast of north Spain may be the result of at least two independent introduction events.

The population source and pathway of introduction of *C*. *dilatata* in Europe are still unclear. Nevertheless, some lines of evidence allow some mechanisms to be proposed. Imports to Spain of fresh *M*. *galloprovincialis* mussels cultured in southern Chilean mussel farms appear as the most likely primary introduction pathway. Although in its native range *C*. *dilatata* may dwell on rocky boulders, kelp hold fasts, and barnacles [32–34, 71, 73, 86, 87], individuals of this species are common epibionts in subtidal mussel beds [33, 52, 57, 88]. In bays heavily polluted by the fishing industry of central Chile, *C*. *dilatata* is a dominant species together with several mytilid species [89] including *M*. *galloprovincialis*, which has been formerly misidentified as the Chilean mussel *Mytilus chilensis* (Hupe 1854) [90]. Secondly, in the introduced region, living *C*. *dilatata* may be found attached to stones and empty bivalve shells (present study). As an epibiont, however, it preferentially lives on wild and farmed *M*. *galloprovincialis* and is only found occasionally as an epibiont on other intertidal molluscs (i. e. *Littorina littorea*) (present study). In O Grove, *C*. *dilatata* was found as a biofouling agent of unattached mussel aggregates scattered on the sea bottom and mussels from raft culture ropes (present study). In El Arbeyal, individuals were found both clinging to clumps of *M*. *galloprovincialis* mussels of marketable size scattered on the sea bottom and to wild native mussels overgrowing artificial substrata; individuals were not, however, found on the cup oyster *Crassostrea sp*. (present study). Collin et al. [52] found individuals attached to mussels close to mussel wharfs in Beluso Bay, a small mussel farming zone in the Pontevedra estuary.

**Fig 12:**
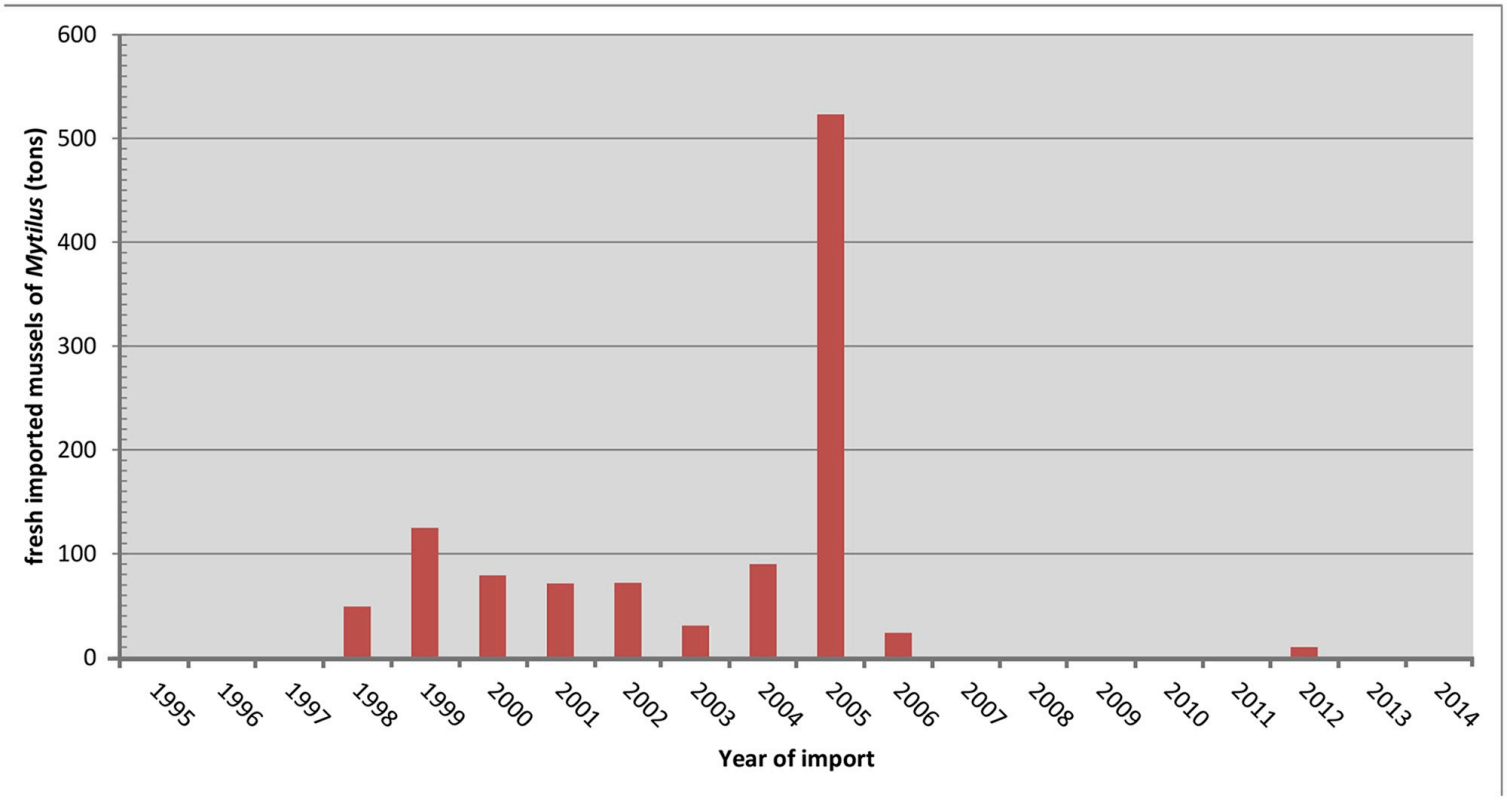
Imports of fresh *Mytilus galloprovincialis* mussels from Chile by Galicia. Source of data from the database Datacomex [91].

Fluxes of mussel imports (S2 Table, Fig 12) lend further support to southern Chile as the potential source of introduction of *C*. *dilatata* into Spain. Galicia, the region where this species was first recorded, is the main target market of Chilean mussel production and export [56]. In the period between 1998 and 2006, Galicia imported annually between 10 and 532.02 metric tons of fresh mussels (*M*. *galloprovincialis*) from Chile for its canning industry. Interestingly, imports peaked in 2005 (S2 Table, Fig 12) coinciding with the year in which *C*. *dilatata* was first recorded live in Galicia. Fresh mussel imports were then interrupted for four consecutive years (2007–2011) and reinitiated in 2012 (Fig 12). In contrast, there are no records of fresh mussel imports from Argentina for the same period (S2 Table). An unnoticed primary introduction of *C*. *dilatata* from Chile to other importing countries, Italy, Netherlands, United Kingdom, Ireland and Denmark (see S2 Table), followed by secondary dispersal from any of these countries to Galicia is less parsimonious and unlikely to have occurred. The volume of fresh mussels imported by these countries for the same period is negligible compared to the amount imported in Galicia. Italy, as the second main market of fresh mussels from Chilean farms, imported a total amount from 2007–2011 that represented less than 25% of the peak volume imported by Galicia in 2005 alone (S2 Table).

Independent primary introductions via transoceanic mussel importation from Chile directly to Gijon is unlikely. During the period from 1999 to 2014, the Principality of Asturias, the autonomic community to which Gijon pertains, did not import fresh mussels from Chile. Assuming that *C*. *dilatata* is really present in the Ebro Delta, what has to be definitively confirmed, this introduction mechanims into this site is also highly unlikely, because Catalonia, the autonomous community of the Ebro Delta, imported only a negligible amount (ca. 11 metric tons) of mussels once, in 2002 (S2 Table). A secondary dispersal from populations on the NW Atlantic coast of Spain to Gijon and to the Ebro Delta, assuming the real presence of *C*. *dilatata* in the latter locality, is the most likely hypothesis. Given the reduced dispersal capability of *C*. *dilatata* juveniles and pediveligers compared to the planktotrophic larvae of *C*. *fornicata*, secondary dispersal of *C*. *dilatata* from the Rias Bajas to Gijon and to Ebro Delta has almost certainly been mediated exclusively by human activities. In Gijon, the most likely human mediated dispersal mechanism is the accidental release during the intraregional trade of fresh commercial Galician mussels. Under this scenario, batches of live *M*. *galloprovincialis* mussels farmed in the Arousa estuary and infested with *C*. *dilatata* would have been transported to the port of El Musel in Gijon, where some mussels may have dropped to the sea during manipulation by buyers and intermediaries. Alternatively, propagules of *C*. *dilatata* may have been released together with effluent waters from conditioning tanks before being sold to the retail market or final consumers. The following evidence supports the above hypotheses. First, according to our results, non-native *C*. *dilatata* uses farmed Galician mussels as dispersal vector and has entered the complex supply chain of fresh commercial Galician mussels from the mussel farms to the final consumer. Second, 98% of the national mussel production is generated by Galician mussel farmers, and around 75% of their mussels is harvested in the Arousa estuary [55]. Third, regardless of the low sample size used here, identical COI gene haplotypes found in all *C*. *dilatata* specimens from O Grove and Gijon point to producers from the Arousa estuary as the main supplier of fresh mussels contaminated with the slipper limpet.

Considering that the Ebro Delta is an important shellfish production centre and the second largest *M*. *galloprovincialis* mussel culture region in Spain [108], and assuming the presence of *C*. *dilatata* in this locality as certain, we propose that any of the following activities may contribute to a hypothetical secondary dispersal of *C*. *dilatata* from Galicia to the Ebro Delta: a) transplantation of mussel seeds produced on floating Galician nursery rafts to mussel farms in the Ebro Delta for growth to commercial size, b) transplantation of ripe mussels from Galician rafts to rafts in the Ebro Delta to restock mussel farms, and c) transplantation of marketable size mussels to the Ebro Delta to satisfy consumer demands. Various lines of evidence support these proposed secondary dispersal mechanisms. Mussel farms in the Ebro Delta occasionally face problems of low production due to red tides, high mortalities of farmed mussels during the summer months, and/or scarce seed supply [108]. In situations of low seed production, mussel farms in the Ebro Delta depend on seed supply from other mussel production zones and even import seeds from Italy and France [108, 109]. Galician mussel farms lead the national mussel production and thus supply mussel seeds from nursery rafts, adult mussels for restocking, and marketable mussels to the Ebro Delta during periods of low production. In addition, intraregional and interregional spat transplants or the transplantation of ripe individuals among different shellfish farms for restocking or fatting is a common shellfish aquaculture activity [5, 108, 110–112] that is also practiced by mussel farmers [41, 110, 113]. Such intraregional movements of commercial molluscs among shellfish farmers have also been considered as the dispersal mechanism leading to the geographical spread of other non-native species with direct development, for example *Ocinebrellus inornatus* [110, 112] and *Cyclope neritea* [111]. Moreover, the relaying of consigned mussels has been proposed as a dispersal mechanism for another non-native slipper limpet, specifically *C*. *fornicata*, in Ireland and potentially the introduction of this species into Wales [113].

Finally, given the invasiveness of *C*. *dilatata* and taking into account that the shellfish trade and transplantation facilitate the introduction of non-native species as well as the expansion of already established non-native species [5, 9, 29, 110–112], the risk of further expansion of *C*. *dilatata* in the coastal waters of other European countries that are target markets of Chilean and Galician mussels is to be expected. We agree with Wasson et al. [5] in recommending the implementation of policies that protect and enhance the local production and commerce of native mussels and bivalves and that put reasonable limits on the volume and frequency of imported living mussels and bivalves cultured in farms located in biogeographical regions different from those of the target market regardless of whether the commodities imported are native in the target market or not. In addition, sanitary protocols to regularly detect the presence of NIS on farmed mussels as well as on the infrastructure and water effluent of production centres should be applied. Traceability of stock translocations and of fresh traded mussels down to the rafts where they have been harvested is imperative. All these measurements may help to lower the risk of introduction and spread of non-native accompanying species in the recipient region. Local and intraregional transplantations of cultured mussels among mussel farms without biosanitary regulations should be avoided.

## Supporting information

S1 Dataset Data matrix for PCA and k-means cluster analysis. (XLSX)

S2 Dataset Composition of the non-native *C*. *dilatata* aggregations from O Grove and El Arbeyal. (XLS)

S3 Dataset Shell length and sex of non-native *C*. *dilatata* from O Grove and El Arbeyal. (XLS)

S4 Dataset Egg-capsule size of non-native *C*. *dilatata* from O Grove and El Arbeyal. (XLSX)

S5 Dataset Reproductive output of non-native *C*. *dilatata* from O Grove and El Arbeyal. Abbreviations: e = eggs or unshelled embryos in early non feeding stages; j = juvenile; ne = nurse eggs; pv = pediveliger; prevel = preveliger or early feeding stages; ? = female unknown.
(XLSX)

S1 Table Eigenvalues of the principal components and their contribution to the total variance. (DOCX)

S2 Table Fresh *Mytilus spp*. mussels imported from Chile by European countries during the period 1999–2014. Imports by Spain were broken down into the autonomous communities of Galicia, Catalonia, and the Principality of Asturias. Data from Datacomex [91].
(DOCX)

S1 Appendix Anatomy of non-native *C*. *dilatata* from O Grove and El Arbeyal. (DOCX)

S1 Fig Anatomy of non-native *C*. *dilatata*. (a) Dorsal view of a mature female. (b) Dorsal view of the head-foot of an intersex stage. (c) Ventral view of an intersex stage. Abbreviations: c = ctenidial filaments; cg = capsule gland; cm = columellar muscle; ct = cephalic tentacle; dg = digestive gland; dm = dorsal mantle muscle; f = foot; nl = neck lobe; o = ovary; os = osphradium; p = penis; r = radula; s = stomach; sn = snout; sr = seminal receptacle; ss = style sac.
(TIF)

S2 Fig Egg capsules of non-native *C*. *dilatata* with pediveligers and nurse eggs. (TIF)

S3 Fig Linear relationship between average female size and the midpoint of the female size range of *C*. *dilatata*. (TIF)
