# Supplementary material for: The alien slipper limpet Crepipatella dilatata (Lamarck, 1819) in northern Spain: A multidisciplinary approach to its taxonomic identification and invasive biology
Source: PLoS One. 2018 Oct 30;13(10):e0205739. doi: 10.1371/journal.pone.0205739 (PMC6207300; doi:10.1371/journal.pone.0205739)
Supplement: S1 Table — (DOCX) [file pone.0205739.s006.docx]

**S2 Table. Eigenvalues of the principal components (PC) and their contribution to the total variance obtained by the PCA.**

| **PC** | **Eigenvalue** | **% Total Variance** | **Cumulative**  **Eigenvalue** | **Cumulative Variance %** |
| --- | --- | --- | --- | --- |
| **1** | **4.315** | **61.65** | **4.315** | **61.65** |
| **2** | **1.536** | **21.94** | **5.851** | **83.59** |
| **3** | **1.033** | **14.76** | **6.884** | **98.35** |
| **4** | **0.109** | **1.56** | **6.993** | **99.91** |
| **5** | **0.003** | **0.04** | **6.996** | **99.95** |
| **6** | **0.002** | **0.03** | **6.998** | **99.98** |
| **7** | **0.001** | **0.02** | **7.000** | **100.00** |
