# Supplementary material for: The alien slipper limpet Crepipatella dilatata (Lamarck, 1819) in northern Spain: A multidisciplinary approach to its taxonomic identification and invasive biology
Source: PLoS One. 2018 Oct 30;13(10):e0205739. doi: 10.1371/journal.pone.0205739 (PMC6207300; doi:10.1371/journal.pone.0205739)
Supplement: S2 Table — Imports by Spain were broken down into the autonomous communities of Galicia, Catalonia, and the Principality of Asturias. Data from Datacomex [91]. (DOCX) [file pone.0205739.s007.docx]

|  | **Fresh imported *Mytilus* mussels from Chile in metric tons** | | | | | | | | | | | | | | | | |
| --- | --- | --- | --- | --- | --- | --- | --- | --- | --- | --- | --- | --- | --- | --- | --- | --- | --- |
|  |  |  |  |  |  |  |  |  |  |  |  |  |  |  |  |  |  |
| **Importer country** | **1999** | **2000** | **2001** | **2002** | **2003** | **2004** | **2005** | **2006** | **2007** | **2008** | **2009** | **2010** | **2011** | **2012** | **2013** | **2014** | **Total 1999-2014** |
| **Netherland** |  |  |  |  |  |  |  | 22.00 |  |  |  |  | 0.10 |  |  | 9.60 | 31.70 |
| **Italy** |  | 25.00 |  |  | 50.00 | 23.00 |  |  |  | 24.00 |  |  |  |  |  |  | 122.00 |
| **United Kingdom** |  |  | 0.20 |  |  |  | 9.30 | 10.00 |  |  | 22.00 |  |  |  |  |  | 41.50 |
| **Ireland** |  |  |  |  | 6.00 |  |  |  |  |  |  |  |  |  |  |  | 6.00 |
| **Denmark** |  |  |  |  |  |  |  |  | 22.00 |  |  |  |  |  |  |  | 22.00 |
| **Spain** | 142.40 | 79.20 | 95.40 | 121.70 | 30.60 | 90.00 | 523.00 | 24.00 |  |  |  |  |  |  |  |  | 1,106.30 |
| **Galicia** | 124.99 | 79.16 | 71.42 | 71.85 | 30.64 | 90.00 | 523.00 | 24.00 |  |  |  |  |  | 10 |  |  | 1,025.06 |
| **Principality of Asturias** |  |  |  |  |  |  |  |  |  |  |  |  |  |  |  |  | 0.00 |
| **Catalonia** |  |  |  | 11.45 |  |  |  |  |  |  |  |  |  |  |  |  | 11.45 |
