# Supplementary material for: The alien slipper limpet Crepipatella dilatata (Lamarck, 1819) in northern Spain: A multidisciplinary approach to its taxonomic identification and invasive biology
Source: PLoS One. 2018 Oct 30;13(10):e0205739. doi: 10.1371/journal.pone.0205739 (PMC6207300; doi:10.1371/journal.pone.0205739)
Supplement: S1 Appendix — (DOCX) [file pone.0205739.s008.docx]

Morphological description

Shell: teleoconch 1.87- 44.1 mm long, without periostracum, compact, rather thick, with the external shell sculpture consisting only of faint, closely packed growth lines. Apex (protoconch), subdistal, smooth, coiled, tightly ad-pressed to a narrow, horizontal extension of the posterior right margin of the teleoconch. Internally, teleoconch with a deep shell muscle impression at the right corner of the shell septum and a faint, small circular dorsal muscle impression medially and at half shell length. In large individuals, home scars left by hitchhikers are frequently visible on the right side of the dorsal shell surface, very rarely on the left side and, occasionally in very large individuals, all over the shell surface. In large individuals (large males, intersexual phases and females), the dorsal surface of the shell acquires a hazelnut brown to brown-violet colour sometimes interrupted by a distinct white narrow, slightly arched zigzagging band extending from the anterior margin of the shell aperture at the level of the food pouch along the shell crest backwards. Internally, the colour of the shell surface varies from uniformly white with a slight violet hue and a dark violet band along the shell margin to uniformly hazelnut brown to dark brown or violet. The shells of juveniles and small males are white on either side, irregularly speckled with brown pigmented flecks which are arranged in numerous, narrow, discontinuous radial bands close to the apex. The shape of the shell is polymorphic and varies among sexes and between sites. Shell septum in all specimens is thin, fragile, white, concave in its left half, somewhat translucent, and extends from the posterior end of the shell to half of the maximum shell length; its free edge is sigmoid, with the curvature at the left side reaching deeply back, thus forming a deep sinus between the shell wall and the left margin of the septum.

Anatomy (S5 A-C): foot broad, half as long as the shell, with a disc-shaped sole projecting into a short, truncated propodium grooved transversally along its anterior edge. Border of the foot sole and base of the foot pigmented with black. Foot sole ventrally and dorsally creamy white. Protruding head with a pair of black well-developed, cylindrical tentacles bearing eyes at half their length (S5 B and C). Head connected to the rest of the body by a dorso-ventrally flattened neck expanding laterally behind the cephalic tentacles into broad, paired neck lobes, the left lobe broader than the right one (S5 B). A longitudinal ridge is present, running from behind the snout along the left side of the neck backwards till the entrance to the pallial cavity (S5 B). Head and neck lobes are dorsally black and ventrally creamy white (S5 B and C). Squat snout extends laterally into angulated projections; in its centre, a large, slit-like, vertical mouth orifice as high as the snout opens to a wide buccal cavity (S5 C). The paired well-developed tubular salivary glands are of variable length. Mantle skirt broad, extending slightly beyond the head and foot sole, creamy white with its edge black pigmented internally (S5 A and C). In individuals from O Grove, the mantle edge is blotched internally with bright yellow pigment. Sickle-shaped, narrow mantle cavity extends from the right side of the head to the posterior tip of the visceral mass (S5 A). The food pouch is placed anteriorly on mantle margin, dorsal to the snout and as wide as the snout. Ctenidium formed by rod-shaped ctenidial leaflets with swollen tips extending from right tip of the food pouch backwards to the posterior tip of the visceral mass. Osphradium morphologically variable, asymmetrical and bipectinate or monopectinate (S5 C), short, brownish, extending from the left tip of the food pouch along the free edge of the mantle backwards to the point where the mantle edge thickens and becomes spongy. Male penis (Fig 3A) dorso-ventrally flattened, with an open, deeply furrowed sperm duct along the ventral mid-line, without papilla but transversely ridged, dorsally black and ventrally white, gradually tapering from the base to the distal end and reducing in size with growth. All females with a small, vestigial, conical or wart-like penis at the end of a ridge running along the right flank of the head and neck.

Visceral mass: reduced, triangular, dorso-ventrally compressed, with its free tip reaching back till the posterior margin of the foot or up to 2/3 its length (Fig 3A and B; S5 A). Gonad, white or orange-coloured in males (Fig 3A), yellow in females (Fig 3B and S5 B), shifted to the left side of the visceral mass, visible dorsally and ventrally. In males, it occupies a narrow stretch along the left margin of the visceral mass, while in mature females it occupies up to 2/3 of the region delimited by the style sac and the stomach, both visible dorsally. Greenish-brown digestive gland divided dorsally by the large stomach and the style sac into an anterior and posterior region, the latter extending between gonad and stomach (Figs 3A and B; S5 B). Ventrally, it flanks either side of the straight posterior oesophagus which opens ventrally into the posterior region of the stomach. Stomach projects anteriorly into the style sac, which curves immediately back to the left running parallel to the long pericardium. Intestine continues ventrally along the style sac in anterior direction towards the kidney looping back before continuing into the rectum (Figs 3B and S5 B). Rectum opens in a protruding anal papilla somewhat behind the mantle edge. Pericardium and kidney enclosed by the ascending and descending loop of the intestine. In males, seminal vesicles ventral to the digestive gland at the anterior right corner of the visceral mass. Columellar muscle circular in cross section, massive, short, situated between right anterior corner of the visceral mass and mantle edge, extending from foot dorsally to the shell (Figs 3A and B; S5 B). Additional weak, slightly bilobed, dorsal shell attachment muscle present, superficial to the kidney (Fig S5 B). Capsule gland placed slightly anteriorly and to the left of the shell muscle, banana-shaped, with distinct lobes divided by transverse septa continuing posteriorly into a short tubular pale pinkish albumen gland to which open into four roundish seminal receptacles (Fig 3B; S5 B). Female genital pore opens on top of a blunt cylindrical female papilla detached from the mantle, deeply grooved eccentrically and longitudinally.

Radula (Fig 3C): taenioglossate radula (2+1+R+1+2) with a powerful central tooth flanked on either side by one lateral and two marginal teeth. Rachidian tooth trapezoidal, with its base slightly broader than its cutting edge, the latter projecting into a prominent central lancet-shaped main cusp flanked bilaterally by three pointed secondary cusps. Lateral tooth much larger than rachidian, triangular, with the internal margin of the shaft shorter than the outer margin and in right angle with the broad base. Ventral surface of the lateral tooth slightly bent with a shallow knot in the centre to accommodate the inner marginal tooth. Cutting edge triangular, protruding in a central, lancet-shaped main cusp flanked at its base by 1-2 strongly reduced subdistal secondary cusps at its inner and outer margin. Outer margin of the lateral tooth below the subdistal cusps serrated, with 5-10 lateral lancet-shaped secondary cusps diminishing in size towards the base of the tooth. Internal margin of the lateral tooth below the subdistal secondary cusp with a larger lancet-shaped secondary cusp. Marginal teeth sickle-shaped, the inner one broader than the outer marginal. Inner edge of the inner marginal presenting 6-11 secondary cusps, while outer edge bearing 5-9 secondary cusps. External edge of the outer marginal smooth. Internal edge of outer marginal presenting 2-4 small secondary cusps at half of the length of the tooth.
