# Supplementary figures and images for: The alien slipper limpet Crepipatella dilatata (Lamarck, 1819) in northern Spain: A multidisciplinary approach to its taxonomic identification and invasive biology
Source: PLoS One. 2018 Oct 30;13(10):e0205739. doi: 10.1371/journal.pone.0205739 (PMC6207300; doi:10.1371/journal.pone.0205739)

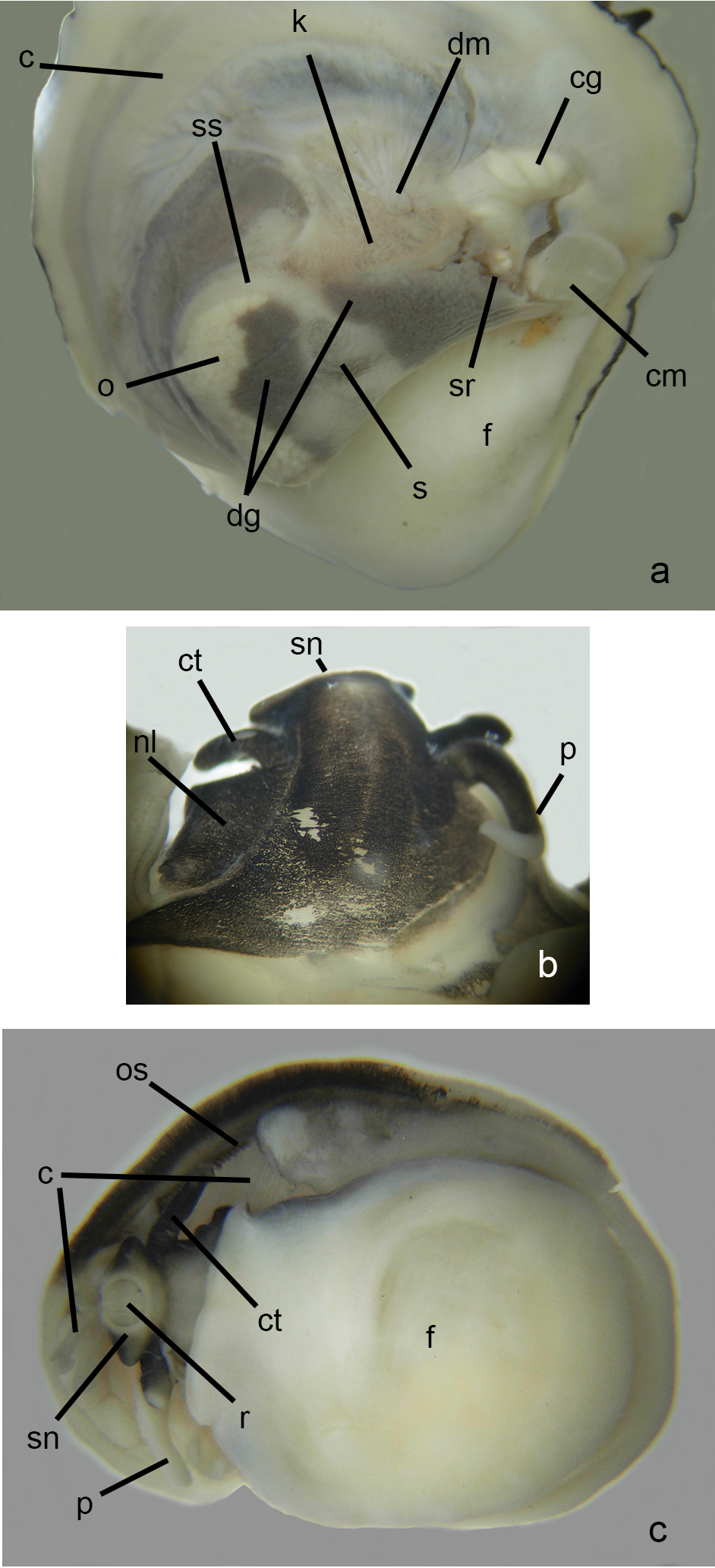

Supplement: S1 Fig — (a) Dorsal view of a mature female. (b) Dorsal view of the head-foot of an intersex stage. (c) Ventral view of an intersex stage. Abbreviations: c = ctenidial filaments; cg = capsule gland; cm = columellar muscle; ct = cephalic tentacle; dg = digestive gland; dm = dorsal mantle muscle; f = foot; nl = neck lobe; o = ovary; os = osphradium; p = penis; r = radula; s = stomach; sn = snout; sr = seminal receptacle; ss = style sac. (TIF) [file pone.0205739.s009.tif]

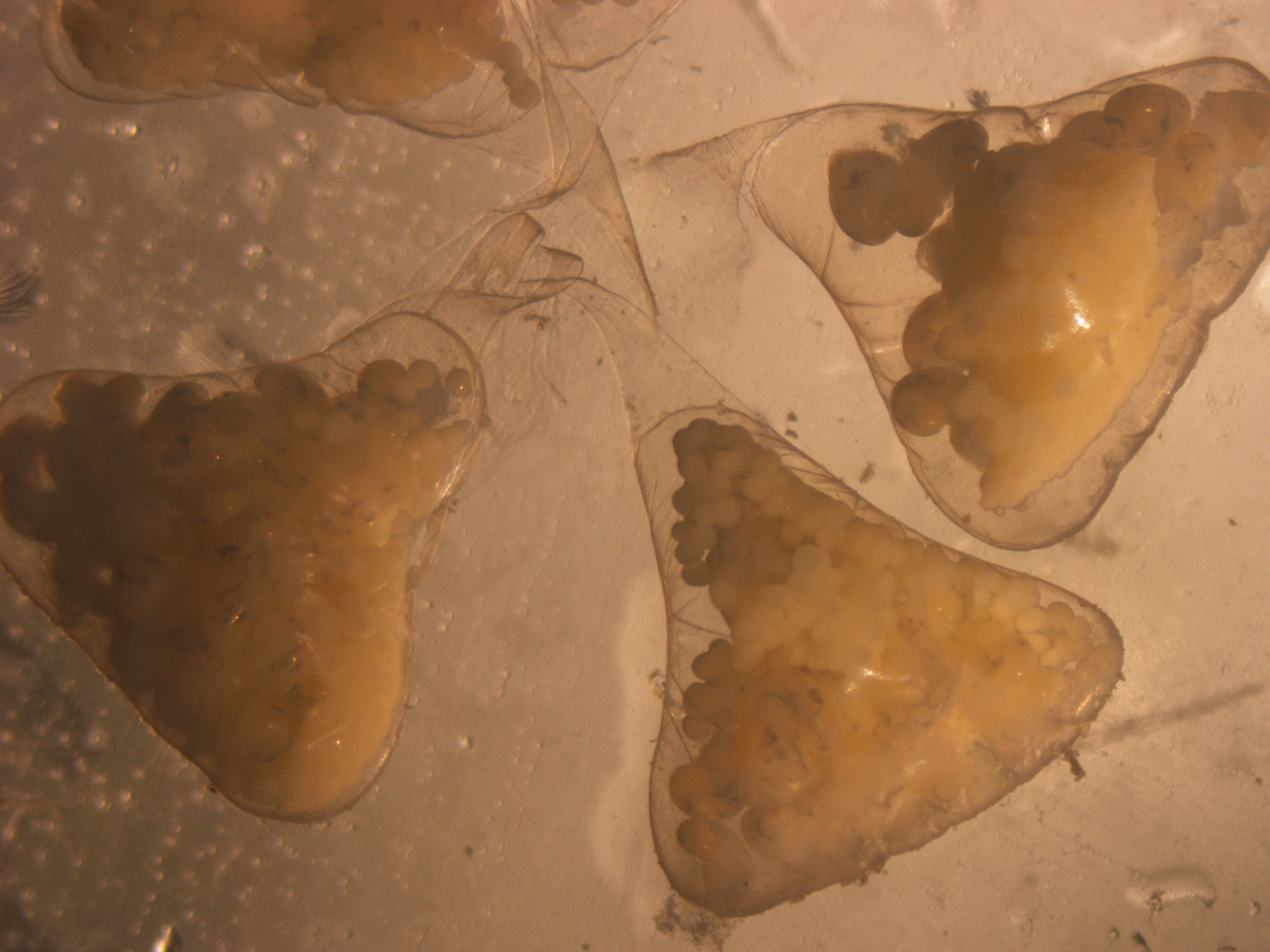

Supplement: S2 Fig — (TIF) [file pone.0205739.s010.tif]

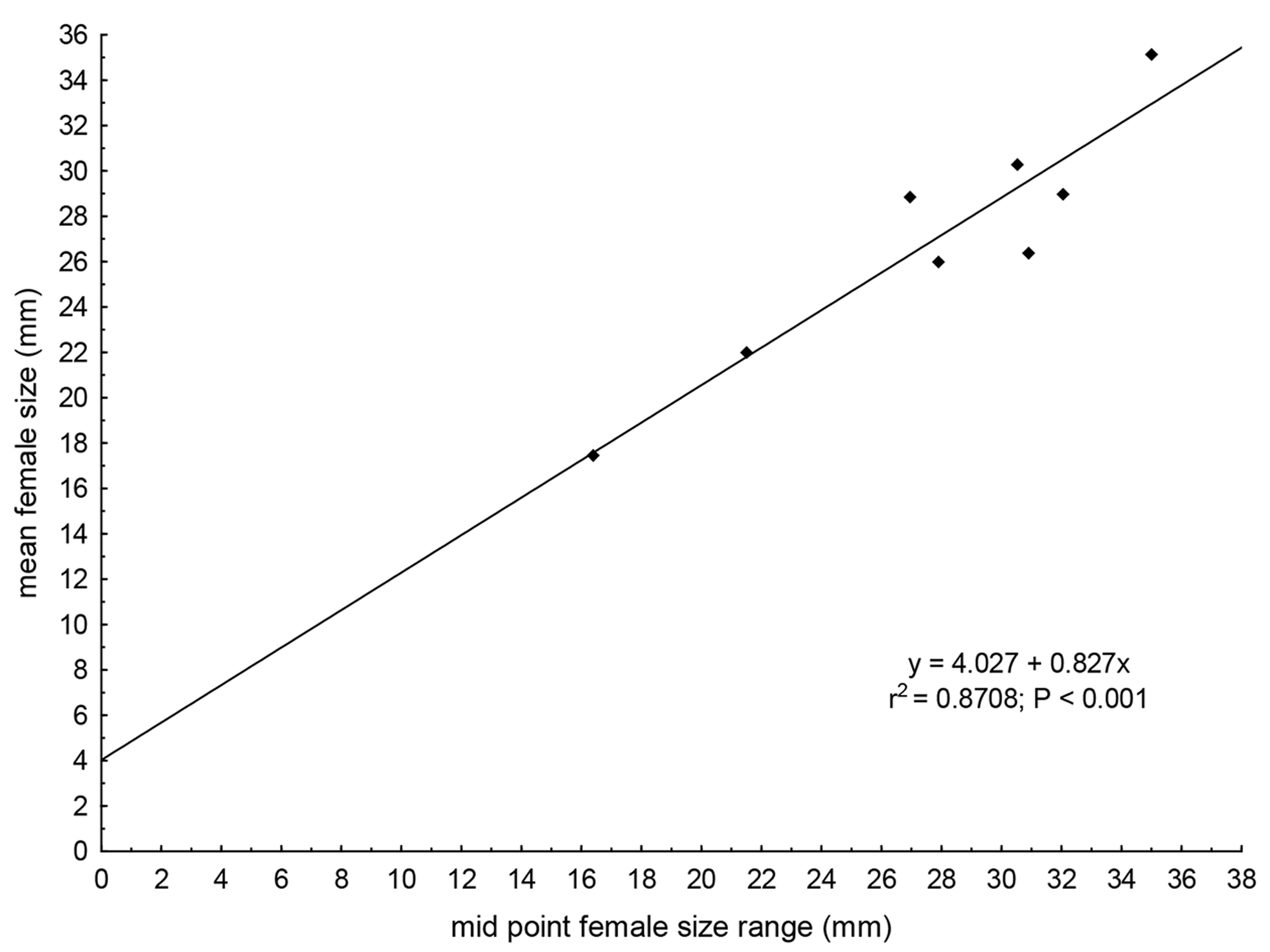

Supplement: S3 Fig — (TIF) [file pone.0205739.s011.tif]
